# Litho-structural mapping using remote sensing, petrographic characterization, and SEM/EDX mineralization analysis: insights from the Arabian-Nubian Shield

**DOI:** 10.1038/s41598-026-52191-6

**Published:** 2026-05-12

**Authors:** Hamada El-Awny, Ahmed M. Abdel-Rahman, Mohamed A. Abd El-Wahed, Hamed G. Hamed, Wael Fahmy, Anas El-Sherif, Árpád Csámer, Ali Shebl

**Affiliations:** 1 Geology Department, Faculty of Science, Al-Azhar University, P.O Box 11884, Nasr City, Cairo, Egypt; 2 Department of Geology, Tanta University, Tanta, 31527 Egypt; 3 Egyptian General Mineral Resources Authority (EMRA), 11517 Abbassia, Cairo, Egypt; 4 Engineering College, Northern Border University, Arar, Saudi Arabia; 5 Department of Mineralogy and Geology, University of Debrecen, Debrecen, 4032 Hungary; 6 Cosmochemistry and Cosmic Methods Research Group, University of Debrecen, Debrecen, 4032 Hungary

**Keywords:** Harga Zarga, Lithological mapping, Remote sensing, Eastern Desert, Gerf nappe, Egypt, Environmental sciences, Solid Earth sciences

## Abstract

This study conducts a thorough geological assessment of the Gabal Harga Zarga (Hadal Darjah) district in the southern Eastern Desert of Egypt using an integrated approach that combines advanced remote sensing, petrographic, and structural techniques. By leveraging hyperspectral PRISMA, Sentinel-2, and PlanetScope satellite data, the research achieves substantial improvements in lithological and structural mapping of an area previously represented as a single metavolcanic unit. The findings reveal a diverse array of lithologies—such as serpentinite, talc-carbonate rocks, metabasalt, and monzogranite—highlighting pronounced tectonic and metamorphic modifications. Structural analysis reveals multiple phases of deformation and underscores the significant influence of NW-SE sinistral faults on hydrothermal fluid flow and associated mineralization. Field validation, together with SEM-EDX mineralogical analyses, confirms the occurrence of economically important minerals, including pyrite, galena, chalcopyrite, silver, bismuth, chromite, zircon, apatite, and molybdenite. These results not only redefine the geological understanding of the area but also point to its significant potential for polymetallic mineralization. The multidisciplinary approach presented here serves as a practical model for mineral exploration and tectonic analysis in other remote and understudied parts of the Arabian-Nubian Shield.

## Introduction

The Arabian Nubian Shield (ANS) is a key location for Neoproterozoic crustal growth in accretionary orogens, serving as a testing ground for new hypotheses on the roles of juvenile crust additions and reworking of crust^1^. The Arabian-Nubian Shield (ANS) represents a significant geological unit composed of juvenile Neoproterozoic rocks found in northeastern Africa and the western part of the Arabian Peninsula. It is the largest contiguous block of juvenile Neoproterozoic crust on Earth^2^. Formed over more than 450 million years from roughly 1000 Ma to 530 Ma—the ANS records a complex crustal evolution tied to the supercontinent cycle involving Rodinia and Gondwana^2–4^. Its formation began with the rifting of Rodinia, which triggered intraoceanic arc magmatism in the Mozambique Ocean^4–9^.

The Arabian-Nubian Shield (ANS) serves as a natural laboratory for studying how upper and lower tectonic plate assemblages are formed and preserved within accretionary orogens. In the far southern reaches of the South Eastern Desert, the Allaqi-Heiani belt and Gabal Gerf massif represent critical segments of the researchYanbu-Onib-Sol Hamed-Gerf Allaqi-Heiani (YOSHGAH) suture zone, as established by previous geological studies^4,10–18^.

The ANS evolved through episodic convergence, arc accretion, syn-tectonic intrusions, metamorphism, and volcanoclastic sediment deposition^7,8^. It is separated from the Saharan Metacraton (central Egypt and Sudan) by a sheared boundary. However, Ediacaran granitoids in the eastern Metacraton point to a major Neoproterozoic reactivation of Archean–Paleoproterozoic crust^3,4,12,19,20^.

The integration of remote sensing has revolutionized contemporary geoscientific investigations, serving as a primary methodology for lithological characterization, structural mapping, and mineral deposit identification e.g.^4,15–18,21–23^. Remote sensing technology has become essential for modern geoscience studies, enabling precise, rapid mapping of lithological units and their associated structural features worldwide^24^. The success of lithological discrimination lies in the fact that all rock minerals exhibit distinct spectral features in remote sensing images due to their unique compositional and structural characteristics^23,25^.

These advanced, multi-sensor remote sensing techniques facilitate fast, cost-effective regional geological mapping, providing a powerful and reproducible methodology for mineral exploration and tectonic studies in the challenging terrains^26–28^.

Improved spectral, spatial, and radiometric resolutions in satellite imagery have been produced thanks to recent developments in sensor technology^23,29^. Modern multispectral and hyperspectral images and techniques provide a streamlined approach to mapping diverse lithologies and hydrothermal alteration minerals, such as iron oxides, clays, sulfates, and carbonates^30–32^. Numerous studies have shown the possible application of remote sensing approaches for mapping REE-Enriched Carbonatite utilizing HYMap data^33,34^, chromite deposits^35^, Albitized granite^36^, post-orogenic granitic intrusions and felsite dikes^37^, Ophiolitic serpentinites and their associated talc carbonates^38^ and mineralized gabbros^39,40^.

In addition to remote sensing contribuations for identifying lithological units and accurate structural analysis is essential for highlighting the primary structural trends. There is limited geological data available for this region, which serves as the primary motivation for this study. Several findings could be considered a novel contribution over the study area. Furthermore, the results may aid in more effective future monitoring and exploration of the area’s potential resources. Additionally, we tried to comprehend the rock units and the geological and structural implications with regard to the regional context of the Wadi Allaqi-Heiani sutures. To better resolve the geological setting of the study area and get a configuration about the lithological units and structural features within the studied terrain. This research implemented various integrated multi‑sensor remote sensing datasets (PRISMA hyperspectral, Sentinel‑2 multispectral, and PlanetScope high‑resolution imagery) with field investigations and petrographic/SEM‑EDX analyses to produce an updated and validated geological map of the Harga Zarga area. This investigation provides several novel and regionally important contributions it considers a first integrated remote sensing‑petrographic‑structural study of the Gabal Hadal Darjah (Harga Zarga) district. The area has been largely overlooked in previous regional maps, which simplistically depicted it as a single metavolcanic block. Our work reveals a more complex lithological mosaic (serpentinite, talc‑carbonate, metabasalt, monzogranite) with significant tectonic imprints. Elucidate the structural framework and deformation history of the area, with special emphasis on the geometry of the Harga Zarga syncline and its relationship to the Hamisana shear zone and the Allaqi‑Heiani suture to help refine the tectonic evolution of the northern part of the East African Orogen and the final assembly of Gondwana.

## Geological and structural setting

The South Eastern Desert (SED) comprises the southern segment of the Gerf nappe and is characterized by a complex assemblage of ensimatic ophiolites, island arc sequences, and syn- to late-tectonic granitoids. Despite their prevalence, these ophiolitic nappes remain understudied and geologically poorly understood due to the logistical challenges of accessing such remote terrain. The southern boundary of the SED is marked by the Nubian portion of YOSHGAH suture zone^10^. They are found along major collision zones e.g.^10,41,42^. Neoproterozoic ophiolites are abundant in Egypt’s SED, with most seeming highly dismembered. However, complete exposures, such as Wadi Ghadir ophiolites, have been observed, as reported by various researchers^43,44^. These comprise sheeted dykes, mafic massive and pillowed lavas, layered and isotropic gabbros, and serpentinized peridotites, with or without a sedimentary overlay of pelagic sediments. Ophiolites are metamorphosed into lower greenschist and amphibolite facies, with serpentinized peridotites often transformed into talc carbonate, and quartz-carbonate bodies along shear zones^45–49^. Numerous studies have examined the ophiolites of the CED e.g^23,46,50^, while few studies have been conducted on the ophiolites of the SED e.g.^23,51^.

**Fig. 1:**
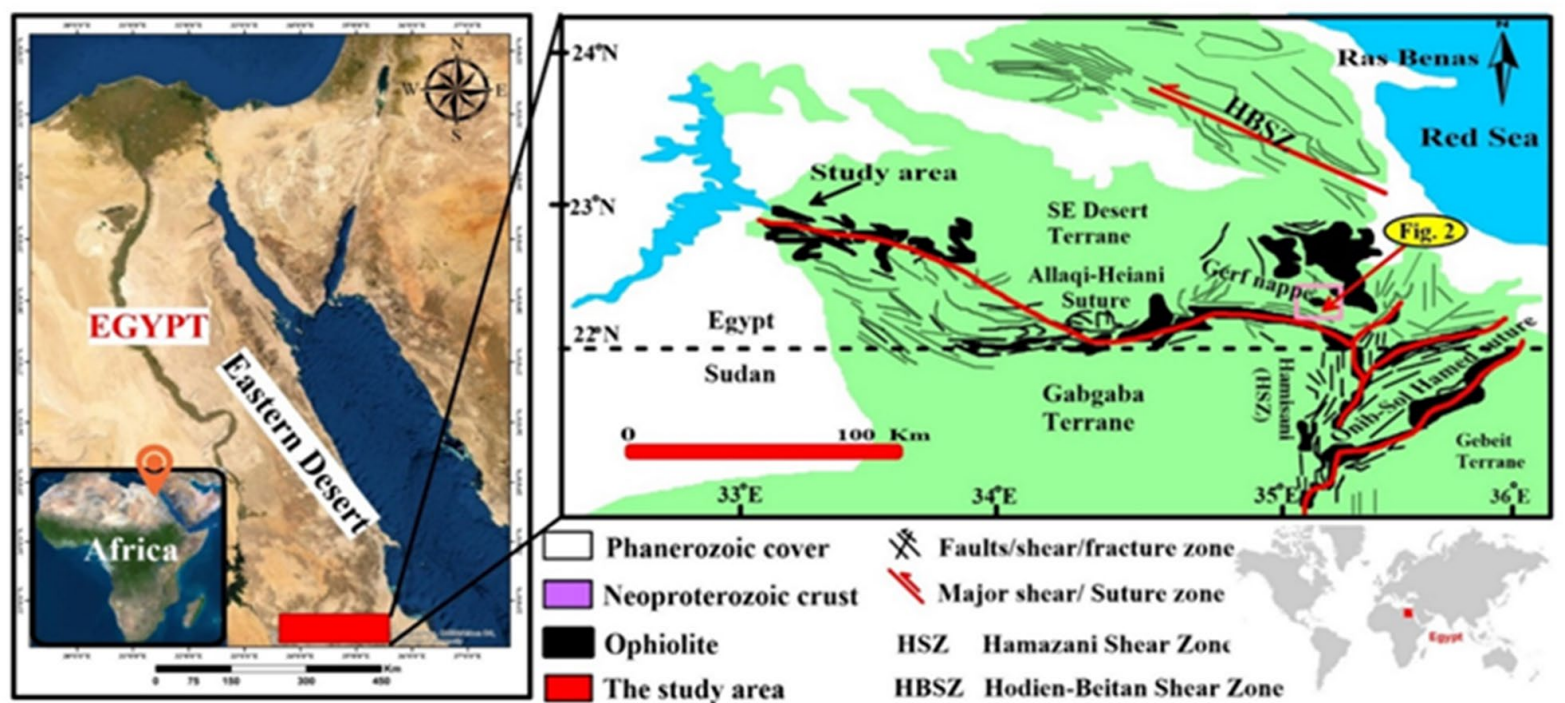
Location map of the studied area with structural features in the southern part of the Egypt, compiled from^65,80,81^. NED: North Eastern Desert, CED: Central Eastern Desert, and SED: South Eastern Desert.

Extending E-NE across the central Nubian Shield, the Allaqi-Heiani suture represents the western portion of an ophiolite-rich fold-and-thrust belt that connects the Nile Valley to the Red Sea coast (Fig. 1). At its midpoint, the suture is overprinted and folded by the N-trending Hamisana shear zone, causing a dextral offset. It continues east of the Hamisana shear zone through the Onib and Sol Hamed ophiolites and into the Arabian Shield near Yanbu in Saudi Arabia. The Heiani area’s ophiolitic rocks, including serpentinized ultramafic rocks, carbonate, talc carbonate, layered gabbro, and pillowed metabasalts, are tectonically connected to surrounding volcaniclastic metasediments. The suture zone within the Nubian Shield serves as the boundary between the South Eastern Desert terrane (~ 750 Ma) to the north and the Gabgaba and Gebeit terranes (830–720 Ma) to the south. This structural interface is defined by ophiolitic sequences of diverse ages, all predating the eventual closure of the Mozambique Ocean^12,13,52^.

The Allaqi-Heiani suture (AHS) serves as the western continuation of the Allaqi-Onib-Sol-Hamed-Yanbu Suture (AOSHY). This geological feature plays a significant role in understanding the tectonic framework of the region, as it delineates the boundaries between different geological formations and provides insights into the processes that shaped the Earth’s crust in this area. The AOSHY suture is predominantly aligned in an east-west direction, exhibiting a significant inclination towards the north-turned-western edge of the Arabian-Nubian Shield. This orientation suggests a complex geological history that has influenced the structural characteristics of the region^8,12^. The findings point to a significant dextral movement of the AOSHY suture in Sudan, which can be linked to the effects of the Hamisana shear zone. This shear zone has played a crucial role in the structural deformation of the region, leading to the formation of a north-trending antiform as the suture folded between 640 and 600 Ma^41,53–55^. The AHS is the only suture in the Arabian-Nubian Shield that showcases a complete ophiolite at Gabal Gerf^41^.

The AHS is characterized by a curved ophiolitic belt that extends over 250 km in the southeastern region of Egypt. The AHS represents a collision zone characterized by an arc-arc interaction, which occurred when the Gerf terrane, located in the northern area (also referred to as the Eastern Desert or Aswan terrane), overrode the Gabgaba terrane situated to the south (∼750 − 720 Ma)^12,56^, before the closure of the Mozambique Ocean (∼ 830 − 720 Ma). The ANS can be categorized into two distinct types of deformational belts. The first category consists of suture-related belts, which are characterized by configurations that involve both arc-arc and arc-continental interactions. These belts are significant in understanding the tectonic processes that shape the region. The second category is comprised of post-accretionary belts, which are marked by features such as compression zones and strike-slip faults that trend northward^8,52^. These geological structures play a crucial role in the ongoing deformation and evolution of the Gabal Harga Zarga area, reflecting the complex interactions that occur after the initial accretionary processes. The presence of arc-arc sutures serves as a key indicator of a collision event involving arc terranes, dated to roughly 800 to 700 Ma.

An east-to-east-southeast trending fold and thrust belt is present in the western Allaqi-Heiani region, characterized by southwest-verging sheets that indicate the existence of a north-dipping subduction zone before the suturing process^12^. According to U-Pb zircon dating of intrusive quartz diorite, island arc assemblages, and ophiolites^42^, suggest that the processes of arc accretion and suture formation took place between 730 and 709 million years ago. Numerous gold-bearing quartz veins are located along the Allaqi-Heiani suture in Egypt’s Southern Eastern Desert, extending over a length of 250 km and a width of 50 km^57^.

The geological chronology established for the central region of the Allaqi-Heiani belt indicates a series of significant tectonic events^55,57^. The first deformation phase (D1) was characterized by a compressional regime that resulted in the development of a fold and thrust belt, accompanied by folds and thrusts oriented towards the west-northwest. The second deformation event (D2) involved a subsequent shortening that reconfigured the previously established fold and thrust belt. This reworking process led to the emergence of plunging folds that are oriented to the northwest, reflecting the complex interplay of tectonic forces in the region. The sequence of these geological events highlights the structural evolution of the Allaqi-Heiani belt. The interplay between the D1 and D2 deformation phases underscores the significance of compressional forces in shaping the geological landscape. The D3 deformation phase was characterized by an east-west contraction, which resulted in the development of both vertical and oblique folds. Additionally, this phase gave rise to transpressional strike-slip shear zones oriented in a north-northwest to south-southeast direction. The geological processes during D3 significantly altered the structural landscape, leading to complex fold geometries and shear zone formations. 2. Following the D3 phase, a subsequent tectonic escape mechanism emerged, directing movement predominantly towards the north. This phase was marked by the reactivation of pre-existing thrust faults and sinistral shear zones, indicating a shift in the tectonic regime.

Reference^52^ proposed that the Allaqi-Heiani belt experienced four distinct phases of Neoproterozoic deformation, designated as D1 through D4. The first two events, D1 and D2, are particularly linked to the initial collision processes that involved the northern Gerf terrane and the southern Haya and Gabgaba terranesIn a contrasting view, the D3 and D4 deformations are attributed to the late stages of collisional tectonics. This interpretation conflicts with the findings of^58^, whose analysis of facing directions and folded thrust geometries indicated a distinct northward vergence and top-to-the-north transport.

The AHS exhibits a left-lateral shear sense, as evidenced by the presence of shear band mylonitic foliation, along with the occurrence of mineral and mica fish structures, and the development of S-C fabrics. These geological features collectively suggest a distinct kinematic behavior associated with the deformation processes at play. As a consequence of progressive shearing, the geological record reveals a complex narrative of folding, which includes the emergence of sheath folds that can be categorized as either planar or non-planar. According to the structural classification by^14^, the Allaqi-Heiani belt is divided into three zones. Domain I (West) is defined by NNE-dipping thrust faults and SSW-verging folds, while Domain II (Central) features upright, isoclinal folding with a characteristic NNW–SSE orientation.This domain also features transpressional faults, which suggest a significant degree of horizontal stress acting upon the geological formations. The geological characteristics of the eastern domain (III) are marked by open folds that trend from north-northwest to south-southeast.

The Gabal Gerf region comprises a metamorphosed ophiolitic sequence alongside an assemblage of metasediments, volcaniclastics, and syn- to late-tectonic granitic intrusions^8,41,59,60^. Notably, the Allaqi-Heiani Suture is distinguished as the only structural boundary in the Arabian-Nubian Shield (ANS) to preserve a fully intact ophiolite at Gabal Gerf^41^ While the Gerf nappe is dated to approximately 750 Ma, the Onib ophiolite to the east reflects an older history, potentially reaching 810 Ma^12,41,42,61^.

The Older Metavolcanics (OMV) of the South Eastern Desert (SED) are widely distributed across several key outcrops, including Shadli, Abu Dahr, Wadi Beitan, and the Gerf and Haimur regions, among others. These formations have been the subject of extensive research, notably by^51,62,63^. In the southern sector of the desert, these metavolcanics are primarily associated with ophiolitic complexes. Characterized by substantial sequences of massive and pillowed tholeiitic basalts overlying feeder sheeted dykes, the OMV is interpreted as a remnant of an ancient oceanic floor formed at a mid-ocean spreading center.

Situated approximately 400 km southeast of Aswan, the Gabal Harga Zarga district lies in the extreme southern reaches of Egypt’s South Eastern Desert (SED). It occupies the southern segment of the Gabal Gerf ophiolite nappe, which stands as the most expansive ophiolitic complex in the Egyptian Precambrian shield, positioned centrally between the AHS and OSH suture zones. Tectonic convergence along the AHOSHY is defined by intense folding, thrusting, and steep imbricate reverse faulting, driving the Gerf arc southward over the 830–720 Ma Gabgaba terrane. Furthermore, widespread southward transport of arc metavolcanics and ophiolites has been documented across various SED sites north of the AHOSHY boundary^64^.

Very scanty geological information is available in the area. The present authors, however, pursued a detailed investigation to understand the rock units, and geological and structural implication with respect to the regional setting of wadi Allaqi-Heiani sutures. This area lies at the intersection of the northern Hamisana Shear Zone (HSZ) and the Allaqi-Heiani Suture Belt, represents a wedge of broken ophiolitic rocks combined with granitic rocks. Talc-carbonate and metabasaltic units constitute part of the most extensive tectonically dismembered ophiolite complexes located near the Red Sea, to the south-southwest of Gabal Gerf (Fig. 1)^65–68^. Harga Zarga is one of the common metamorphic outcrops in the SED^59,69^. The biggest ophiolite occurrence in the ANS that appears to be intact is the Gerf nappe^41,59,70^.

## Materials and methods

### Remote sensing datasets

Several remote sensing datasets were integrated in the current research with different spatial and spectral resolutions. These datasets include Sentinel 2 (up to 10 m), PRISMA (30 m) and PlanetScope (3 m) data. The main aim behind this integration is to analyze the whole area in a regional sense to identify the major structural features and lithological units then get closer to our main focus (Hagar Zarqa) to better characterize it. Sentinel-2 is a broad-coverage, high-resolution multispectral imaging mission initiated by the European Space Agency (ESA) under the Copernicus Program. This mission encompasses 13 spectral bands, offering spatial resolutions ranging from 10 to 60 m. The application of Sentinel 2 data in geological applications has proved to be useful in recent years.

Launched in March 2019 by the Italian Space Agency (ASI), the PRISMA (Preecursore IperSpettrale della Missione Applicativa) satellite operates from a sun-synchronous orbit at an altitude of 615 km. The system captures hyperspectral data across 240 bands within the VNIR (400–1010 nm) and SWIR (920–2505 nm) ranges, maintaining a spectral sampling interval of 12 nm or less. Unlike many other hyperspectral missions, PRISMA includes an integrated panchromatic sensor providing 5 m spatial resolution, which complements the 30 m hyperspectral Ground Sample Distance (GSD) across its 30 km swath^38,71^. PRISMA products are classified according to their specific applications. Level 0 comprises binary files containing instrument and satellite ancillary data (such as cloud cover percentages). Level 1 products consist of radiometrically and geometrically calibrated panchromatic radiance and hyperspectral data cubes. Level 2 processing is categorized into four sub-levels (L2A–L2D), providing progressively refined outputs: L2B offers geolocated ground radiance, L2C provides geolocated reflectance, and L2D delivers both geolocated and geocoded ground reflectance. L2D scene was applied for the current research.

Planet Labs Inc. has consistently upgraded its PlanetScope constellation by phasing out legacy hardware and launching a succession of more advanced sensors. The Planetscope satellite network, which is made up of several launches of separate cubesats known as DOVEs, travels in a sun-synchronous orbit at 98° inclination at altitudes between 475 and 525 km. The evolution of PlanetScope satellite technology encompasses three distinct generations: Dove-Classic (PS-0), Dove-R (PS-1), and SuperDove (PS-2). This study utilizes the SuperDove (PS-2) CubeSat, which provides an eight-band spectral suite including coastal blue, blue, green, green I, yellow, red, red edge, and near-infrared (NIR). The planetscope mission on https://earth.esa.int/eogateway/missions).

### Image processing techniques

Many effective techniques, including false color combination (FCC), band ratio (BR), principal component analysis (PCA), independent component analysis (ICA), and minimum noise fraction (MNF), were used to interpret the lithological and hydrothermal alteration patterns. Displaying spectral characteristics that improve the distinction of rock types in a colored RGB composite image is considered an FCC. It enhances the readability and clarity of images enabling optimal differentiation for the studied rock units. The spectral properties of minerals and rock units are successfully highlighted by the components created by PCA and ICA transformations, which are employed for dimensionality reduction and image enhancement^32^. The dataset can be transformed using PCA, an analytical method that involves aligning the data so that the axis with the largest variance is named the first principal component (PC), the axis with the second-highest variance is named the second PC, and so on. In scenarios lacking prior insight into the data, Independent Component (IC) transformation serves as a valuable method for blind source extraction. Leveraging the non-Gaussian presumption of independent sources, the transformation utilizes higher-order statistics to reveal several features in remote sensing data that frequently exhibit non-Gaussian properties. Even when these notable features constitute only a minor fraction of the image’s pixels, IC adaptation can still differentiate them^72^. ICA was applied in the current research as it has distinct advantages over PCA. IC analysis uses non-Gaussian assumptions and higher-order statistics to reveal features in non-Gaussian datasets, whereas PC analysis depends on orthogonal decomposition and Gaussian assumptions using only second-order statistics. This makes it possible for IC analysis to spot minute details, like abnormalities, that PC analysis could miss due to noise^73^.

Besides PCA and ICA, the Minimum Noise Fraction (MNF) method was also applied to better reveal the data characteristics which may enhance our geological interpretation of the study area. There are two primary steps in the MNF method. It starts by employing a correlation matrix to estimate data noise. Secondly, weighted data packages based on standard deviation are produced by computing correlations within the data^74^. This procedure makes sure that the overall data is kept intact, with the first packages recording surface effects and the last ones include noise.

### Field work and petrographic analysis

**Fig. 2:**
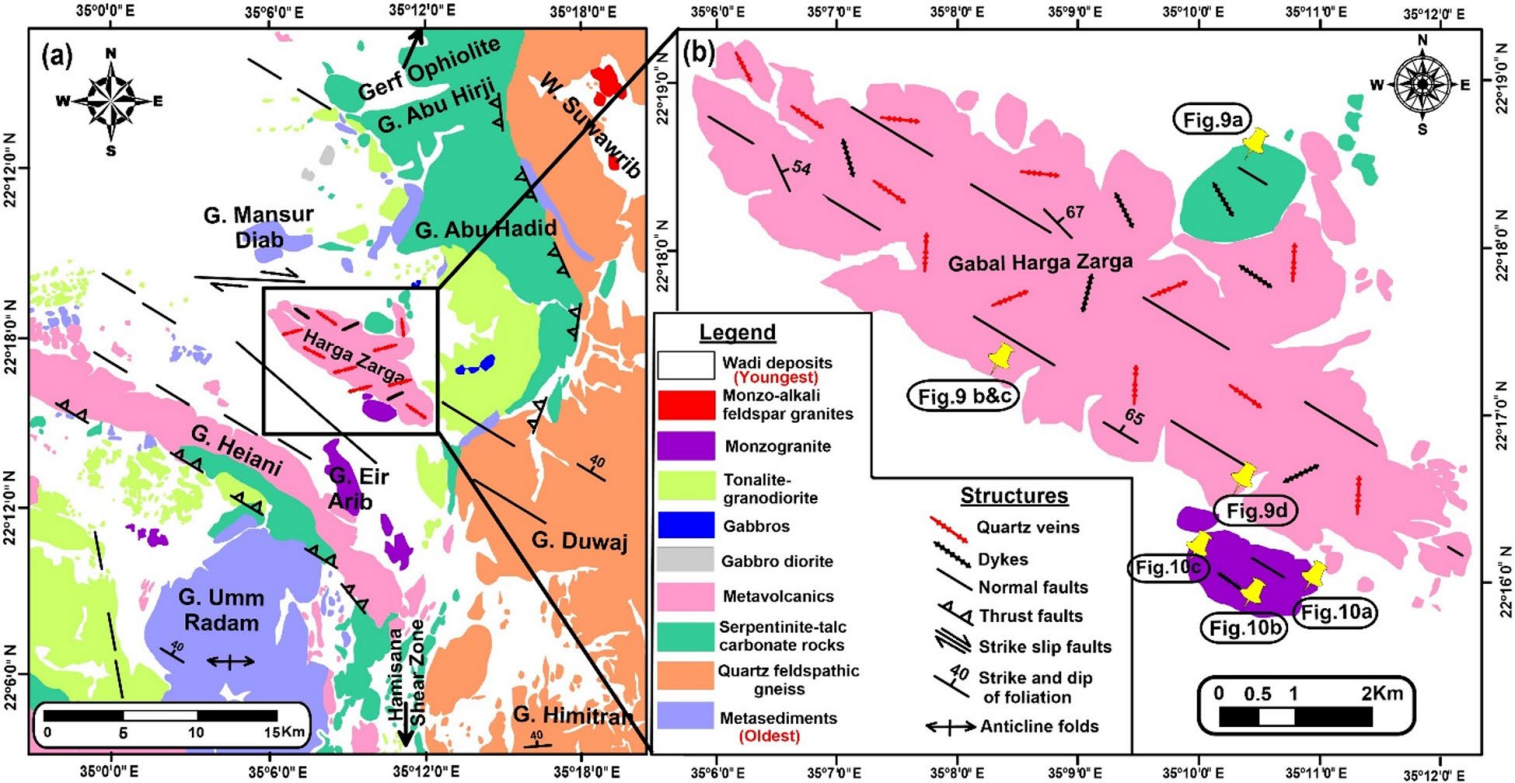
(**a**) Gross lithological map of Harga Zarga above Heiani and Hamisana shear zone, as interpreted from satellite imagery (Sentinel 2 data) and published geological studies modified after^13,41,82–87^. (**b**) Simplified geological map of the district including the location of the field photographs^82,85^.

During the fieldwork, forty representative rock specimens were gathered from the various geological units that cover the region (Fig. 2). Additionally, photographs of noteworthy field findings and structural relationships are included. Within the research region, many structural aspects are examined. Using a polarizing microscope, about twenty-five thin slices from various rock units were examined. These thin slices were examined with a polarizing microscope (Optika B-353POL) at the Department of Geology’s microscopic examination unit at Al-Azhar University in Egypt’s Faculty of Sciences.

### SEM-EDX analysis

The SEM-EDX techniques were applied to thin-polished sections of the studied rock units to characterize ore textures, identify ore minerals, and determine their chemical compositions. SEM-EDX microanalyses were conducted on ore minerals from all investigated samples using a Quanta 250 FEG scanning electronic microscope (Field Emission Gun) equipped with an EDX detector (Energy Dispersive X-ray analyses), at the Central Laboratories of the Egyptian Nuclear Martials Authority (ENMA). The analysis was performed at an accelerating voltage was 30 KV, with magnifications from 14 x up to 1.000.000 x, and a gun resolution of 1 nm.

## Results

### Remote sensing findings

**Fig. 3:**
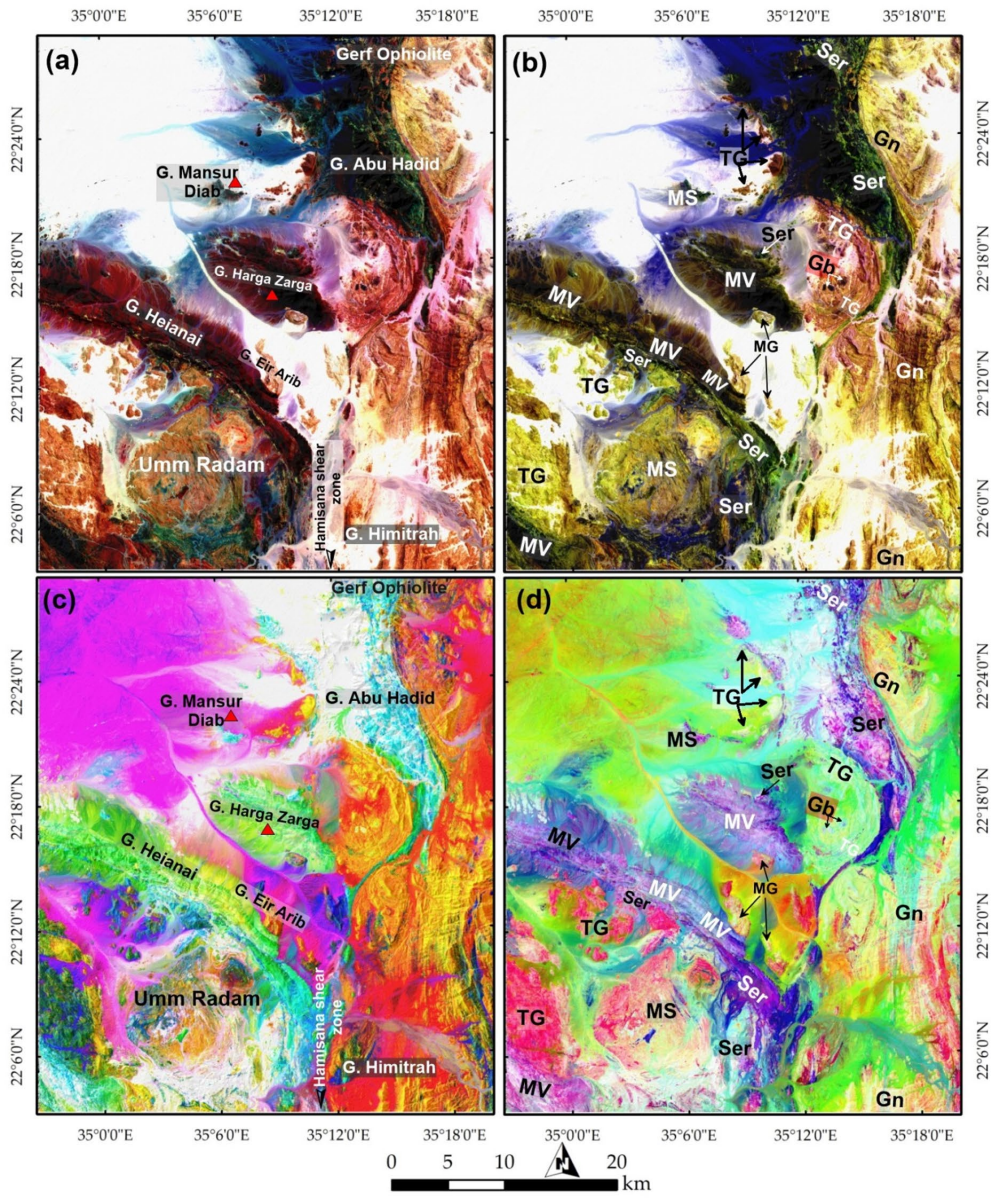
Lithological discrimination using Sentinel 2 data showing the regional tectonic and geological setting of the study area (Hagar Zarqa) utilizing (**a**) FCC 12-6-2, (**b**) FCC 12-11-2, (**c**) MNF 312, and (**d**) MNF 431 in RGB, respectively. Gn; Gneiss, MG; Monzogranite, Gb; Gabbro, MS; Metasediments, Ser; Serpentinites and talc carbonate rocks, TG; Tonalite-granodiorite, MV; Metavolcanics,.

**Fig. 4:**
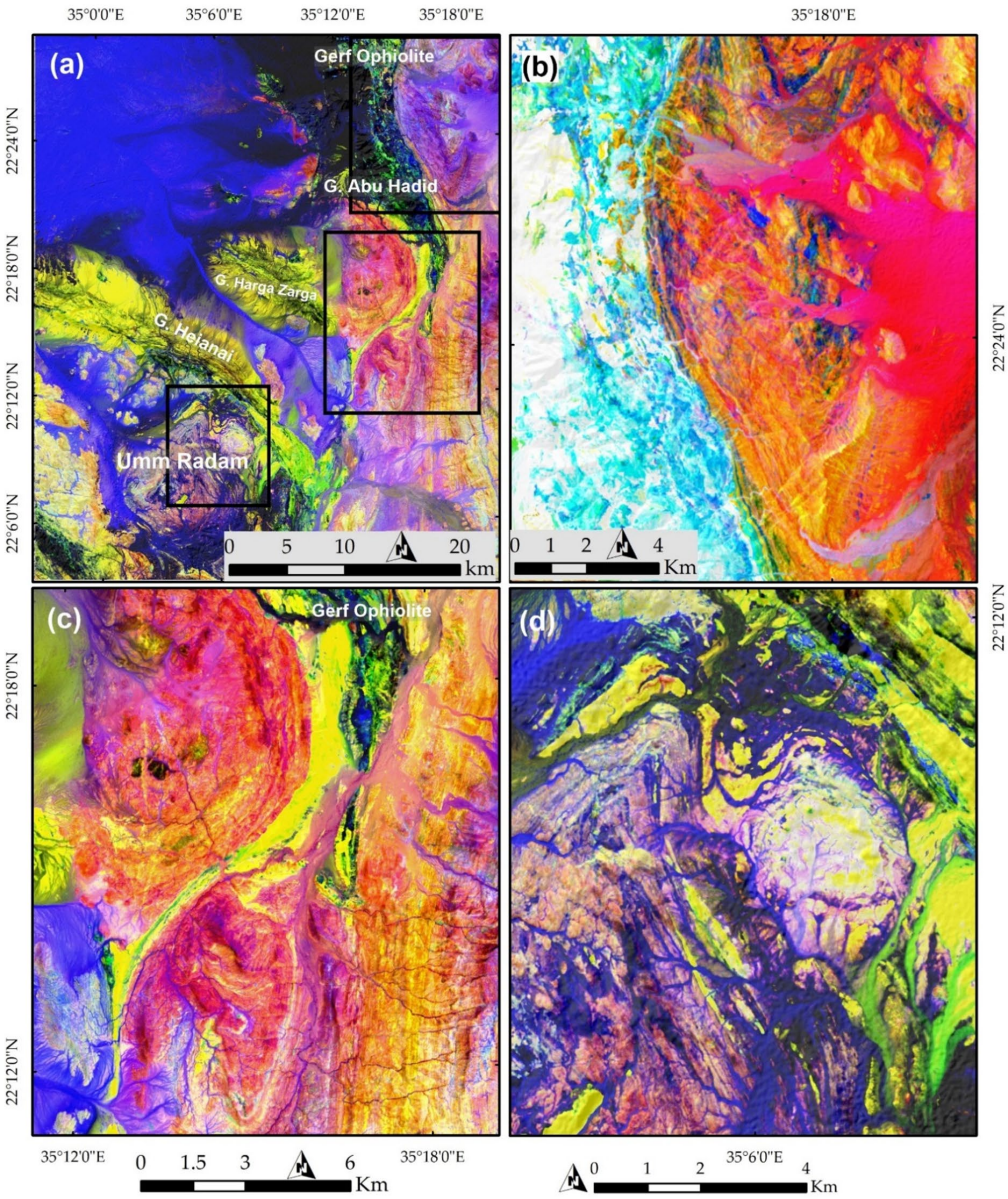
Remote sensing implications, showing (**a**) an overview of the main structures using BR 12/5_11/7_4/2, (**b**) a close-up view of revealing synforms in the northeastern sector depicted by MNF312, (**c**) ductile deformation emphasized by the sheared serpentinite rocks (yellow) in BR 12/5_11/7_4/2, and (**d**) BR 12/5_11/7_4/2, highlighting the prominent ductile structural elements (continued folding) associated with shear zones surrounding the study area.

As stated in the geological setting, the study area is located in the extreme southern part of the Egyptian Eastern Desert and cannot be better interpreted without understanding the surrounding geology. Thanks to remote sensing data covering large areas, we were able to better detect the regional geological settings affecting the study area. Our results show complex structural phases with diverse regimes, predominantly of ductile characteristics, as seen in Figs. 3 and 4. For instance, Fig. 3a–d present different remote sensing combinations highlighting the main lithological characteristics of the studied terrain and its complex structural setting.

Figure 3a–d clearly discriminate quartz-feldspathic gneissic blocks in the eastern part of the study area, represented by West Suwawrib, G. Duwaj, and G. Himitran (light reddish to pinkish colors in Fig. 3a). These figures also highlight the well-known Gerf ophiolitic serpentinites (dark black color at the northern part of Fig. 3a) of G. Abu Hiriji and G. Abu Hadid to the north of the Hagar Zarga study area. Additionally, better discrimination of the G. Umm Radam metasediments block (the oldest subrounded rock unit exhibiting reddish-brown color at the southern part of Fig. 3a) was introduced south of the G. Heiani metavolcanics (exhibiting a dark red color tone as in the Hagar Zarqa area), which is located south of our study area. The northwest corner of the study area is covered mainly by wadi deposits.

Figure 4a–d provide a close-up view of the main lithological boundaries and major structural features (located within the black boxes annotated in Fig. 4a) that may be overlooked in Fig. 3. For instance, Fig. 4b is a close-up view from MNF 312 presented in Fig. 3c, clearly highlighting the sharp contacts between the Gerf ophiolitic serpentinites (west) and the quartz-feldspathic gneissic blocks (east). Additionally, further investigation of the quartz-feldspathic gneissic blocks (reddish) clearly shows several synform structures within the granitic rocks, which can be easily depicted at the extreme northern part. Figure 4a and b present a clear example of the ductility of Gerf ophiolites (shown in yellow) as marked by squeezing between hard rocks in Fig. 4c and multifolding in Fig. 4d.

**Fig. 5:**
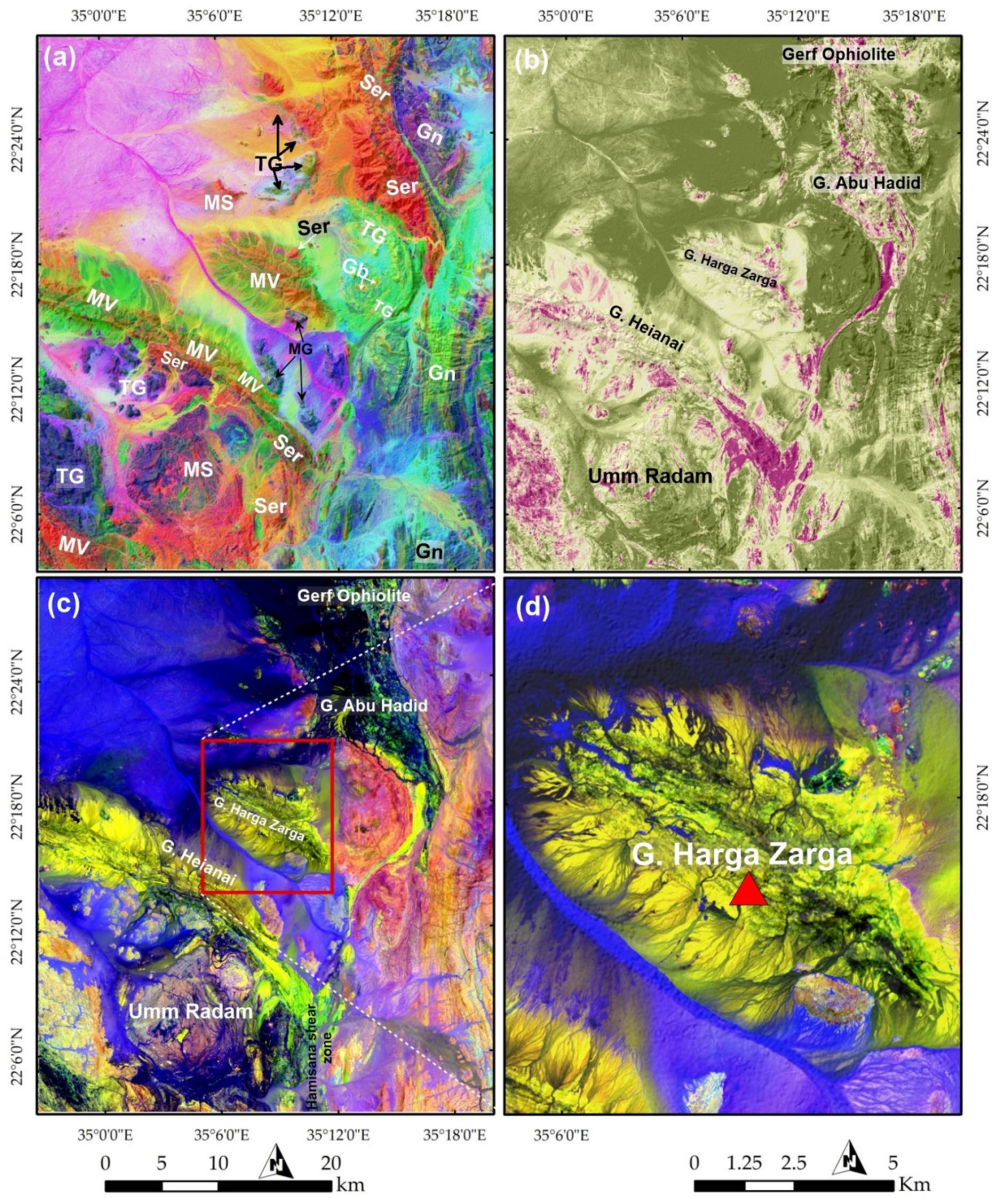
Remote sensing implications showcasing lithofacies discriminations and their structural control, using (**a**) PCA231 in RGB, Abbreviations; Gn; Gneiss, MG; Monzogranite, Gb; Gabbro, MS; Metasediments, Ser; Serpentinites and talc carbonate rocks, TG; Tonalite-granodiorite, MV; Metavolcanics. (**b**) pseudocolor representation of MNF 3, and (**c**) BR 12 5__11 7__4 2 with a highlighted box indicating the location of the Gabal Zarqa area (**d**).

Figure 5a highlights the metavolcanics and ophiolitic serpentinite rocks in green and reddish colors, respectively, and clearly shows a kind of structural branching mostly associated with these rock units, which is depicted in Fig. 5b by pinkish color. Figure 5c clearly shows the lithology (depicted mainly in yellow) and structural similarity (NW trending) of G. Heiani metavolcanics with the Hagar Zarqa study area, presented in Fig. 5d, and will be closely investigated in the following section.

**Fig. 6:**
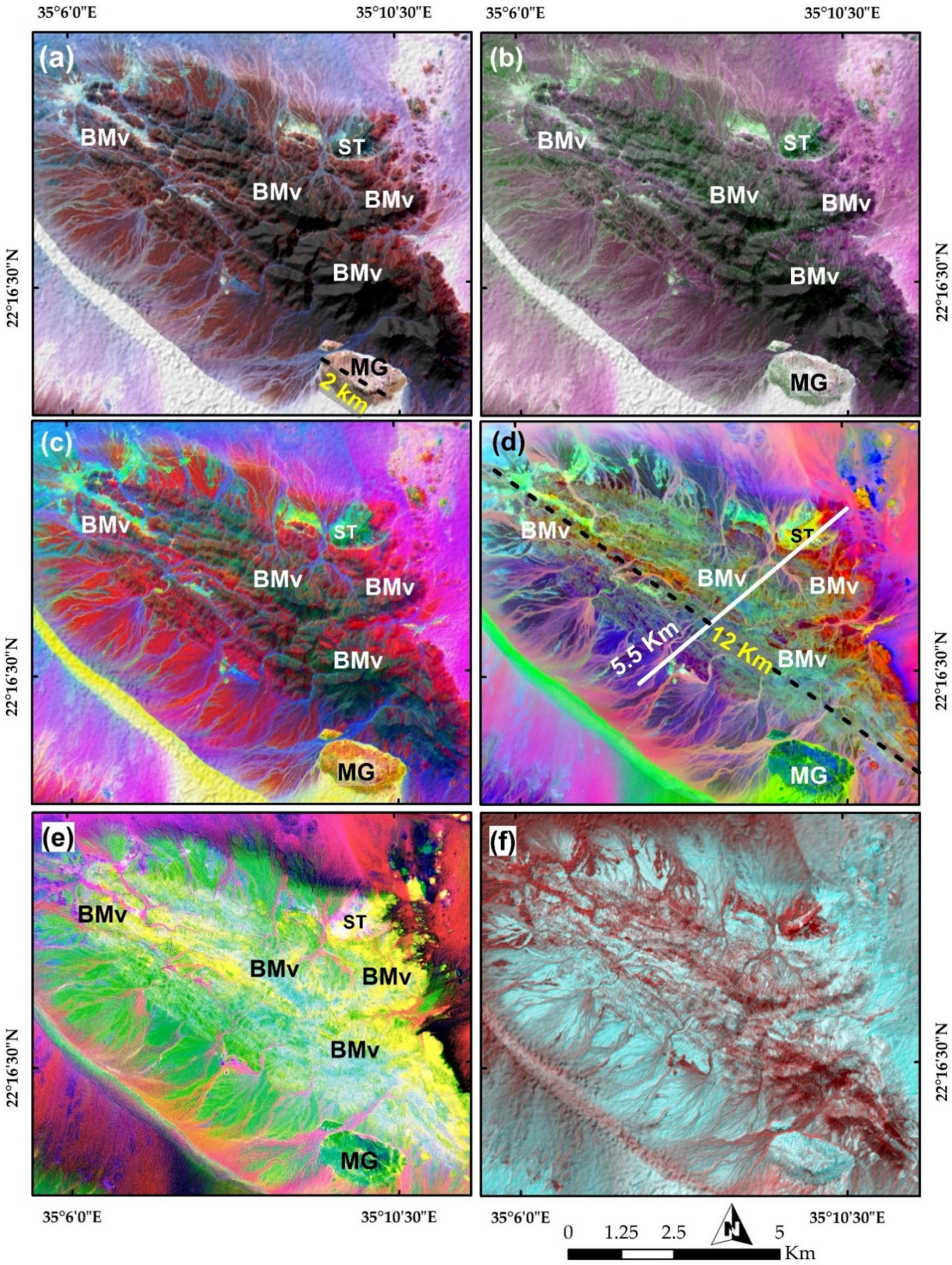
A close-up view of the study area showing Gabal Zarqa and its lithological varieties using the following techniques in RGB format: (**a**) FCC 12-6-2, (**b**) FCC12_4/3_11, (**c**) decorrelation stretch of FCC12_6_3, (**d**) MNF 4-MNF2-MNF3, (**e**) IC 3-IC2-IC4, and (**f**) PC2-PC4-PC2, respectively. MG; Monzogranite (Muscovite granite), BMV; Basic metavolcanics, ST; Serpentinite and talc carbonate rocks.

Figure 6a,b show a generalized view of the Hagar Zarqa area using Sentinel-2 FCC of 12-6-2 and FCC12- 4/3 −11in RGB, respectively. These image combinations clearly show the effect of random mining as bright pixels, especially at the northwestern tip of the studied area. A more detailed lithological differentiation is presented through Fig. 6c,d using decorrelation stretch of FCC 12-6-3 and MNF 4-MNF2-MNF3 in RGB, respectively. Figure 6c clearly discriminates the previously mentioned artisanal mining patches in bright cyanish-green color. Figure 6d highlights lithological variations within the Hagar Zarqa area, depicted by serpentinite and other metavolcanics in brown color within the dominant metavolcanic block of the whole mountain. Additionally, it highlights the monzogranite block in the southeastern part in green color. Figure 6e emphasizes these lithological variabilities with a yellowish color for serpentinite and less-common metavolcanics and cyan color for the main metavolcanic rocks, with a greenish representation at the borders, emphasizing the effects of seasonal showers on the rocks. These observations are confirmed by Fig. 6d, which highlights the various types of drainage networks developed from the center towards the borders of the studied mountain.

**Fig. 7:**
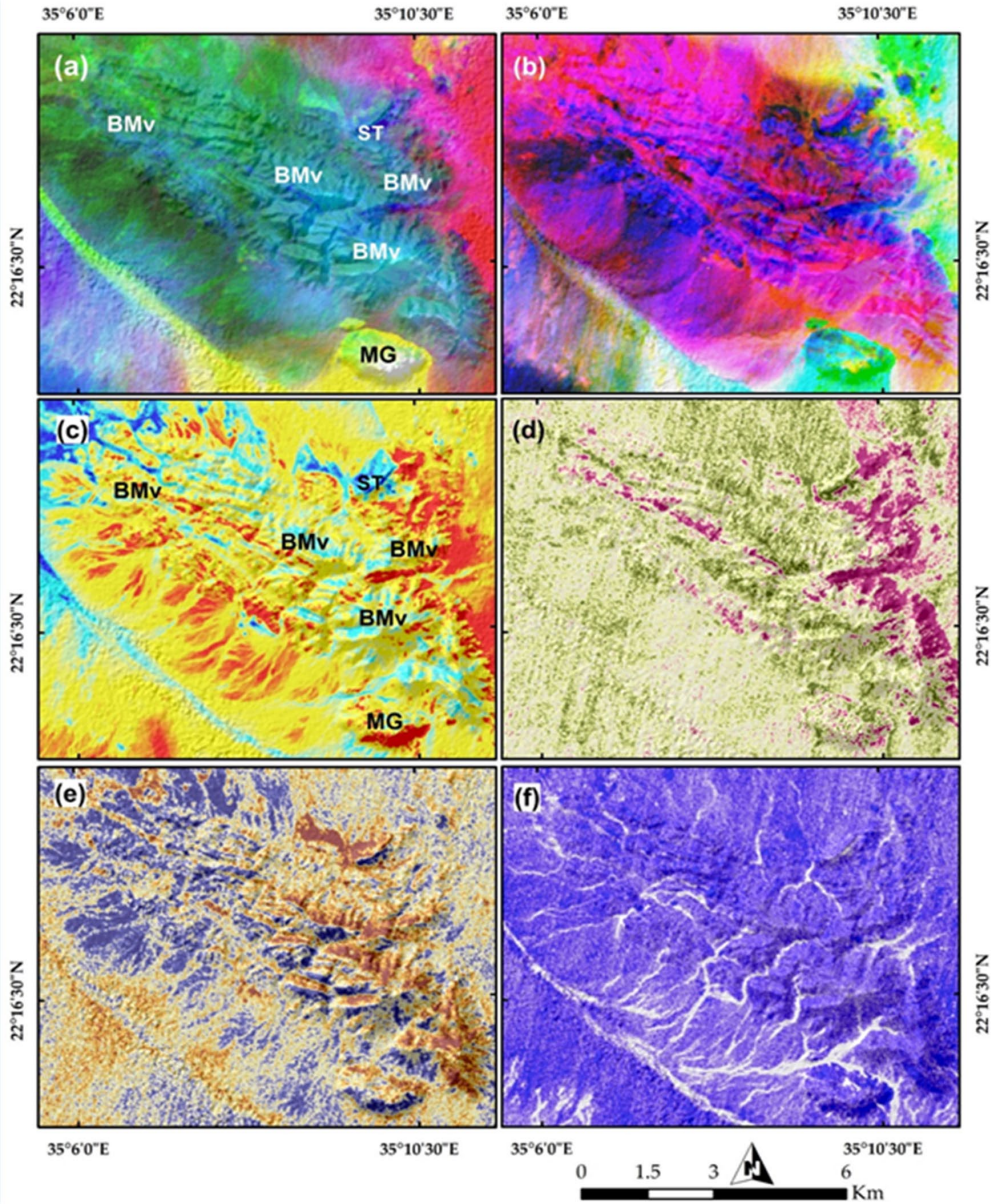
PRISMA combinations showing Gabal Zarqa and its lithological varieties using: (**a**) MNF2-MNF3-MNF4, b) MNF7-MNF2-MNF12, in RGB. Pseudocolor representations of (**c**) PC2, (**d**) IC 11, (**e**) IC12, and (**f**) IC 15. The latter shows the effect of the drainage network on the studied rock units. MG; Monzogranite (Muscovite granite), BMV; Basic metavolcanics, ST; Serpentinite and talc carbonate rocks.

These results highlighted that the study area is not constituted of only a homogenous metavolcanics but another lithological component could be also seen. Towards better discrimination, PRISMA hyperspectral data were applied and highlighted clearly these variations. For instance, Fig. 7a clearly divided the studied mountain into two main lithological blocks the eastern cyan (serpentinite) and the western green (metavolcanics). Figure 7b highlighted some lithological linear extensions of serpentinite and metavolcanics (blue) within the main metavolcanic body. These extensions trending NW-SE highlighting the tectonic control of these rocks. These prolonged rock exposures are separated in Fig. 7c–e. Figure 7f separated the main drainage network of the studied mountain and highlighted how dissected is it.

**Fig. 8:**
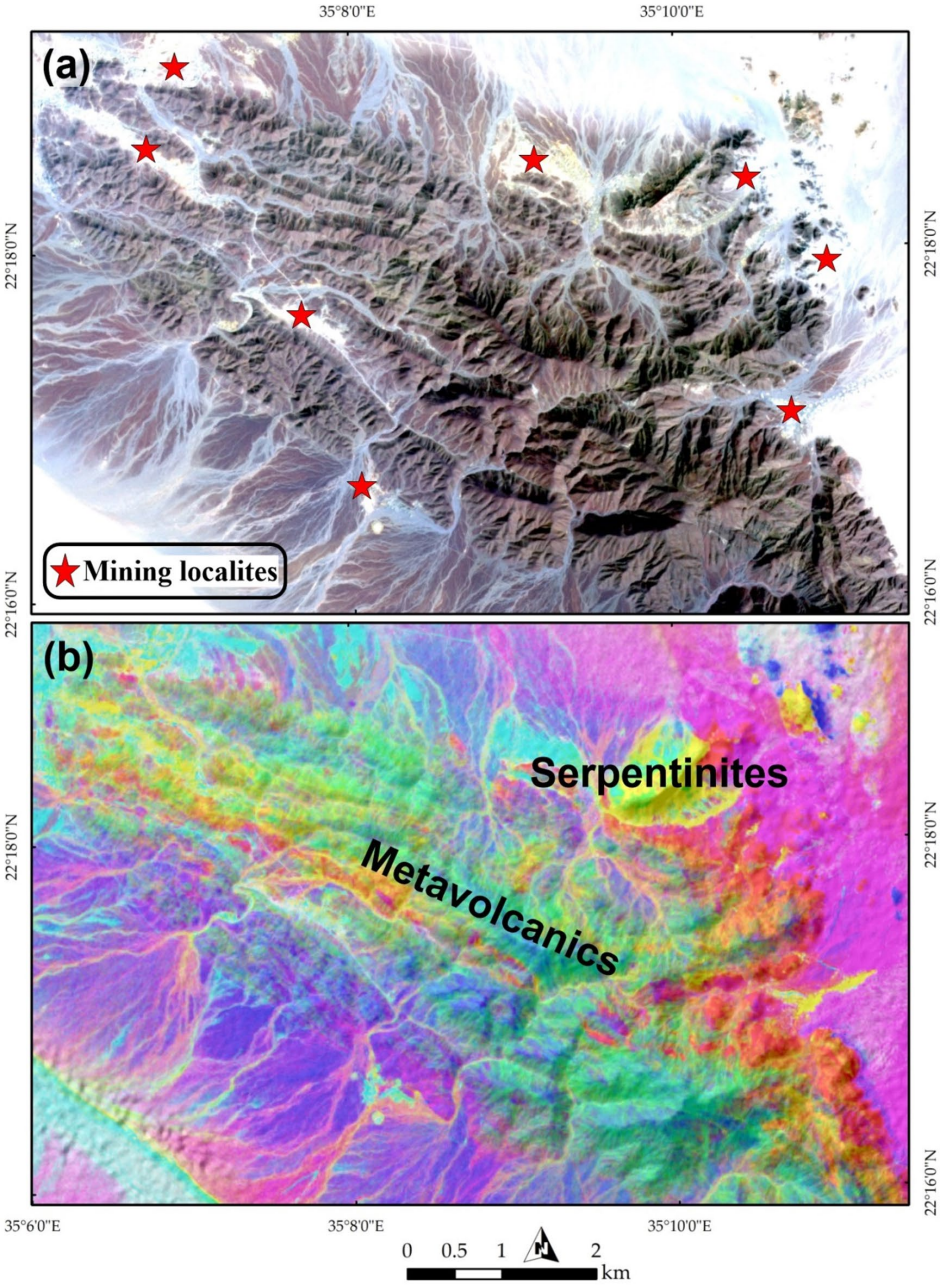
Detailed mapping utilizing 3 m PlanetScope data, showing (**a**) the main mining activities in the study area represented by bright pixels. (**b**) lithological discrimination using a pan-sharpened Sentinel-2 RGB combination of MNF 4-MNF2-MNF3, employing Planet data.

Up to that point, Sentinel-2 and PRISMA effectively highlighted the heterogeneity of the lithological composition of the studied mountain through their spectral information. However, better spatial details can be provided using the 3 m PlanetScope data. Figure 8a presents a pan-sharpened Sentinel-2 RGB combination, enhanced with PlanetScope data, which clearly reveals the details of the mountain. It distinguishes between the eastern coarse block (mainly serpentinite) and the western block (finer metavolcanics) based on texture. Additionally, it shows random mining activities in very bright colors with greater clarity. Further details are provided in Fig. 8b, which uses a pan-sharpened Sentinel-2 RGB combination of MNF 4-MNF2-MNF3, incorporating PlanetScope data. This figure clearly displays minute lithological variations both spectrally and spatially.

### Field validation

The lithological units identified via remote sensing were confirmed through rigorous field validation. The Harga Zarga region consists primarily of metamorphic and magmatic complexes. The metamorphic sequence features ophiolitic serpentinite and talc-carbonate lithologies, alongside basic metavolcanics that locally exhibit pillow structures. These units are cross-cut by a magmatic suite comprising syn- to late-tectonic monzogranite intrusions, as well as various dykes and veins (Fig. 2b).

#### Ophiolitic rocks (serpentinite and related rocks)

**Fig. 9:**
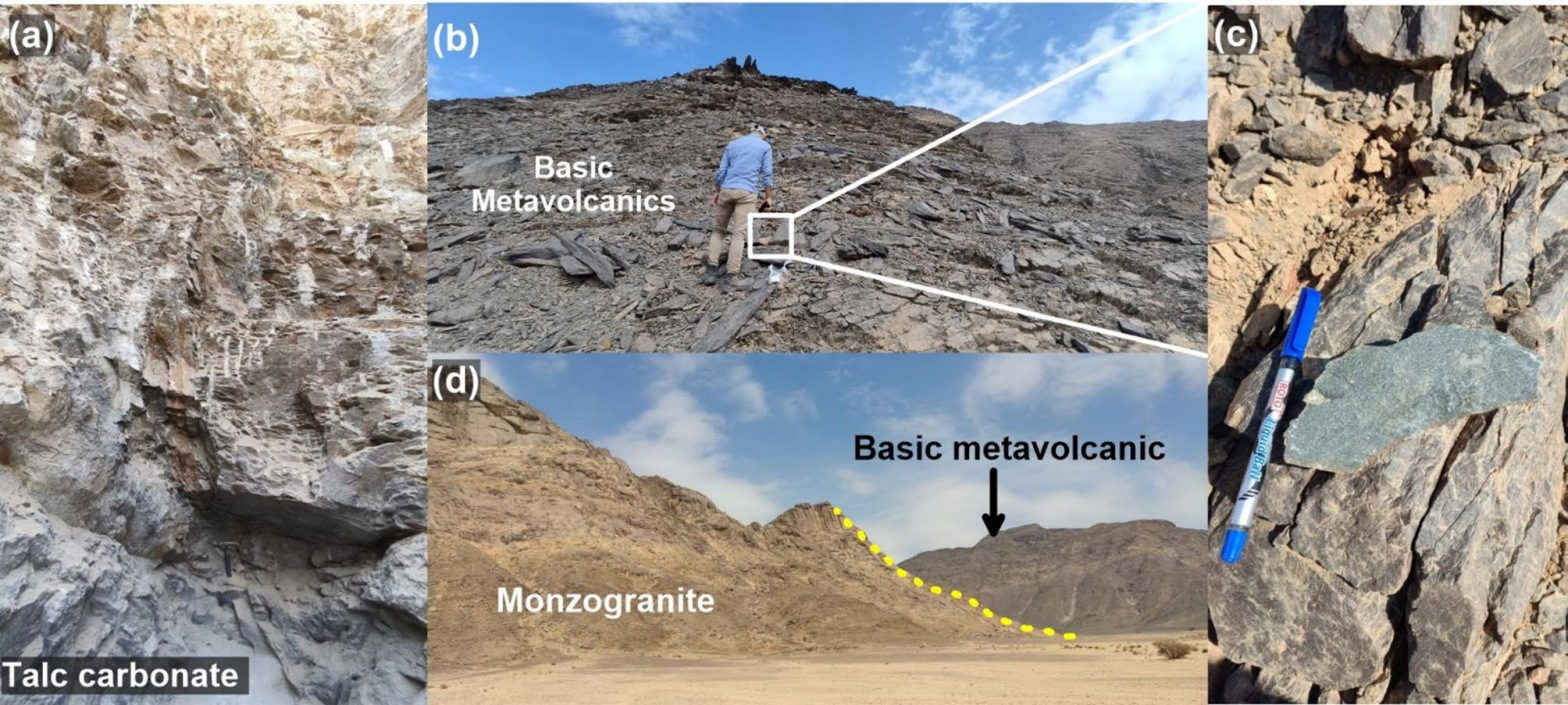
Field photographs show (**a**) Talc-carbonate rock. (**b**) sheared and schistose metavolcanic. (**c**) fine grains of metabasalt. (**d**) Offshoot of monzogranites rocks overlies the basic metavolcanics.

Serpentinite and related talc carbonate rocks are observed in the northern part of the studied areas. Isolate masses of talc–carbonate rocks (0.5–1 km width) along the shear zone between the ultramafic rocks and basic metavolcanic-ophiolitic members are also recorded. Their color is vast, ranging from greenish grey to grey (Fig. 9a).

The metavolcanics establish an extended belt that stretches in an NW-SE direction, covering around 12 km^2^. The most common rock units identified in fieldwork are the basic metavolcanic rocks, which occupy about 45 km^2^ region (Fig. 6). They form thick flows, schistose, massive, fractured and pillowed basaltic composition (Fig. 9b,c). The present ophiolitic is tectonically intruded by syn-tectonic granite^59^. According to^41^, the area is considered the oldest ophiolitic basaltic rocks recorded in the Egyptian Eastern Desert, with an age of 834 million years. The ophiolitic metavolcanics that occur in Gerf-Zarga-Heiani zones are composed of metabasalts and sheeted dykes that have metamorphosed into greenschist facies^41,69,70,75^. According to^76^, undifferentiated lavas and dikes in the carbonated massive and porphyritic basaltic rocks of Gabal Harga Zarga are thought to be a laterally displaced portion of the Heiani or Gerf ophiolitic nappes.

#### Syn -tectonic granitic

**Fig. 10:**
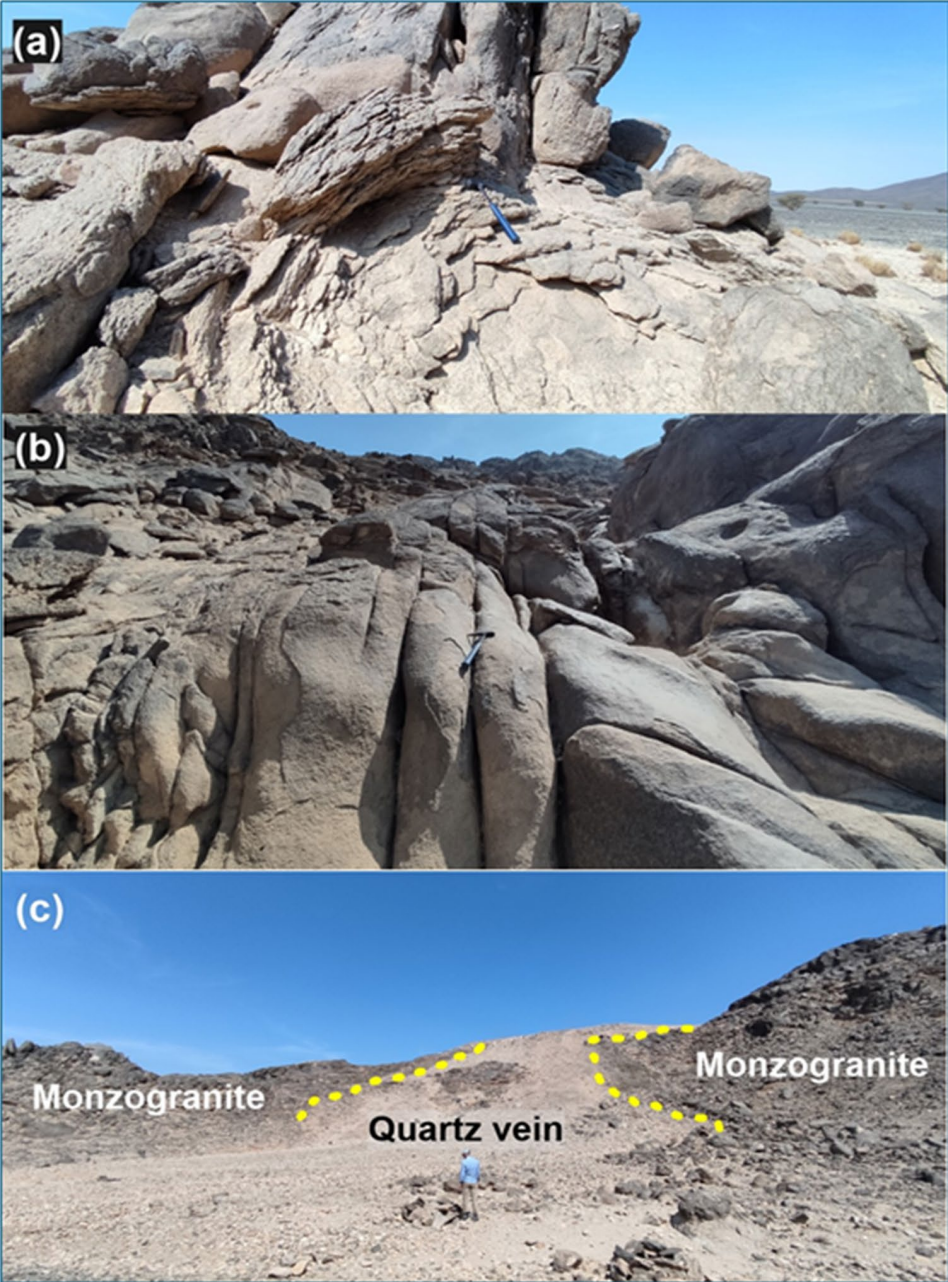
Field photographs show (**a**) exfoliation-affected surfaces (onion shape) with different extension of granitic rocks. (**b**) horizontal jointing in monzogranites. (**c**) huge quartz vein invaded the monzogranites.

The syn-orogenic magmatic intrusions represent low-relief monzogranite and have a dome-shaped structure. It is exposed as a small outcrop south of Harga Zarga complex (~ 2 km, Fig. 6a). They are a medium to coarse-grained, and gray color. It is characterized by exfoliation, highly jointed, and shows highly deformed and highly weathered especially near the contacts (Figs. 9d and 10a,b). These rocks are invaded the basic metavolcanics and intruded by huge quartz veins (Fig. 10c). There are numerous quartz veins of various trends where gold was recently mined.

### Petrographic investigations

The petrographic description of the studied thin sections representing the different rock units that exposed at the study area, which is classified into; talc carbonates, metabasalts, and syn-tectonic granites.

**Fig. 11:**
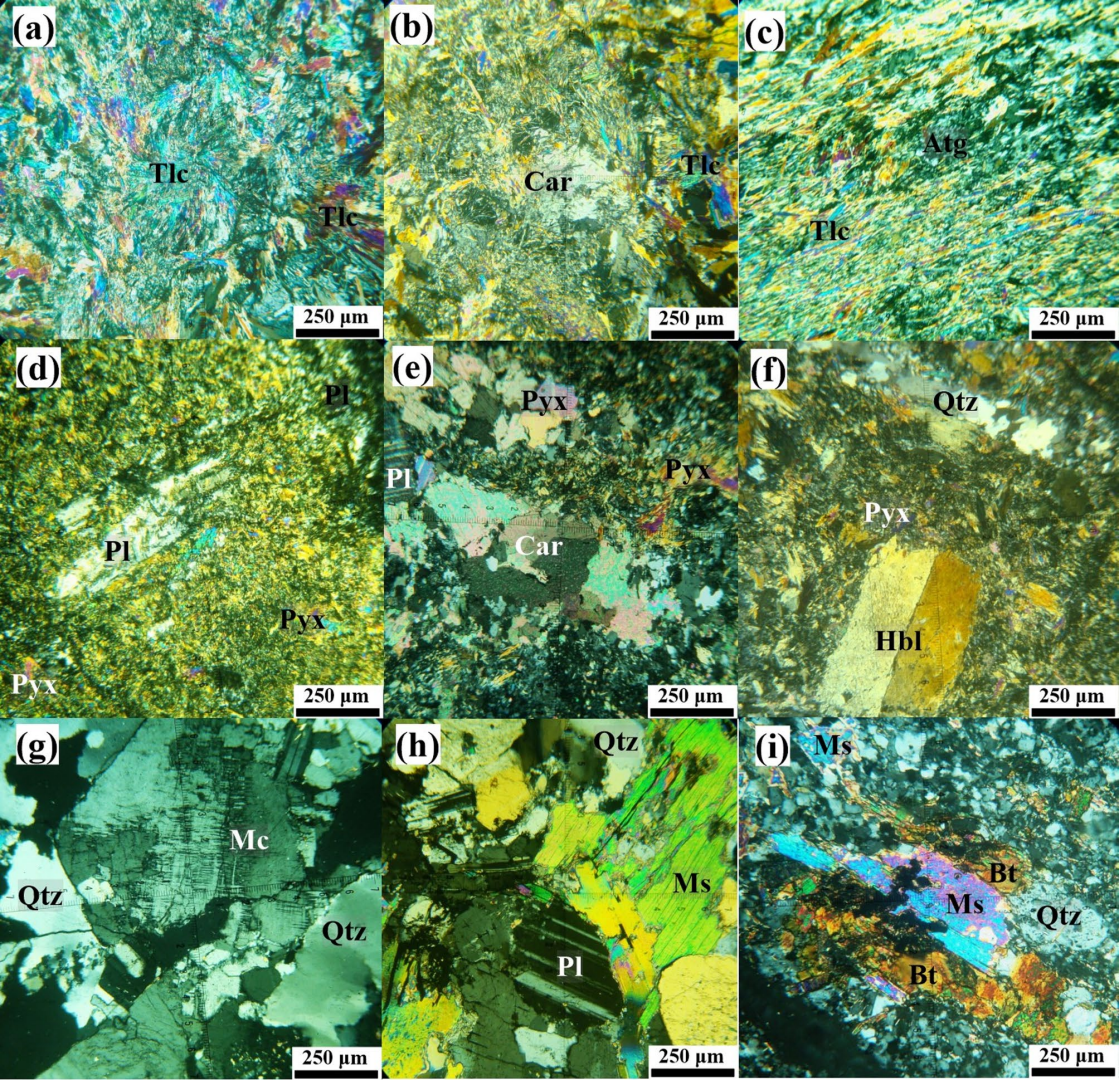
Petrographical features of talc-carbonate, metabasalt, and muscovite granite rocks showing: (**a**) well-developed crystals of talc (Tlc) in talc carbonate rocks. C. N. (**b**) Pocket of carbonate (Car) minerals associated talc (Tlc). C. N. (**c**) Fibrous crystals of talc (Tlc). C. N. (**d**) plagioclase (Pl) lathes surrounded by fine grains of pyroxene (Pyx) in metabasaltic rocks. C. N. (**e**). Microveinlets filled with carbonate (Car). C. N. (**f**) Phenocrysts of hornblende (Hbl) showing baveno twinning with microveinlets filled with secondary quartz (Qtz) C. N. (**g**) Muscovite within quartz C. N. (**h**) Plagioclase enclosed within muscovite crystal C. N. (**i**) Biotite flake association with muscovite and quartz. C. N.

Talc-carbonates in the study area range from dark greenish-grey to light grey or cream-colored. Mineralogically, they consist of balanced proportions of talc, carbonate, and relict serpentine minerals, along with minor opaques. These rocks are predominantly talcose, containing remnants of antigorite and chrysotile, which provide a characteristic creamy, soapy texture. Talc appears as a secondary phase resulting from the alteration of Mg-carbonates (magnesite/dolomite) and silicates like serpentine, tremolite, or chlorite. Microscopically, talc is identified as fine-grained crystals or parallel-stacked platy aggregates with low relief and high interference colors (Figs. 11a,c). Carbonates are observed as distinct clusters, lenses, or veinlets within the talc matrix (Fig. 11b). Additionally, quartz occurs as medium-grained, xenomorphic crystals showing undulose extinction, occasionally forming veinlets that transect the host rock (Fig. 11c).

The metabasalts are characterized as fine-grained, non-porphyritic rocks primarily composed of plagioclase and pyroxene. Secondary alteration has produced chlorite and carbonate minerals, while quartz and opaques serve as accessory phases (Fig. 11d). The groundmass consists of a fine-grained matrix of plagioclase, pyroxene, and hornblende microlites (Fig. 11d). Plagioclase appears as fine-grained crystals, occasionally exhibiting a directive texture, or as larger porphyroblasts; these are frequently altered to zoisite, which obscures their lamellar twinning (Fig. 11d). Pyroxene manifests as subidiomorphic to xenomorphic prismatic crystals that often show significant chloritization along cracks and margins (Fig. 11e). Additionally, fine- to medium-grained carbonates occur as a byproduct of plagioclase alteration (Fig. 11e). Chlorite is the main secondary mineral after pyroxene, characterized by its green color. Quartz present as fine- to medium-, subangular to subrounded grained, disseminated within the rock constituents and sometimes occurs as, veinlets (Fig. 11f).

Muscovite granites consist essentially of potash feldspar, plagioclase, quartz muscovite and biotite with some secondary minerals such as epidote. Potash feldspars occur as large subhedral plates. Perthite exists as colorless, large plates formed from the intergrowth of plagioclase and K-feldspar. It forms different types of perthitic textures (Fig. 11g). Plagioclase occurs as colorless, high relief, subhedral to euhedral platy crystals. Owing to deformation it occurs as large plates of tecoblast crystals and sometiemes show blastic plagioclase crystals. Much of plagioclase crystals are partially to completely altered to sericite and epidote which filling the fractures and the spaces around the crystals (Fig. 11h). Quartz presents as medium to coarse grains and stretched deformed crystals. Interstitially filling the spaces among the other mineral constituents. It exists as colorless, shadow extinction and sometimes exhibits polygonal shape (Fig. 11g). Biotite forms dark brown color, subhedral flakes and moderately dichroic from pale brown to dark brown as well as partially altered to chlorite (Fig. 11i). Muscovite usually associates biotite and opaque minerals and occurs as subhedral flakes dispersed between the essential mineral constituents (Fig. 11h,i).

### Mineralogy of some mineral deposits (SEM/EDX investigations)

**Fig. 12:**
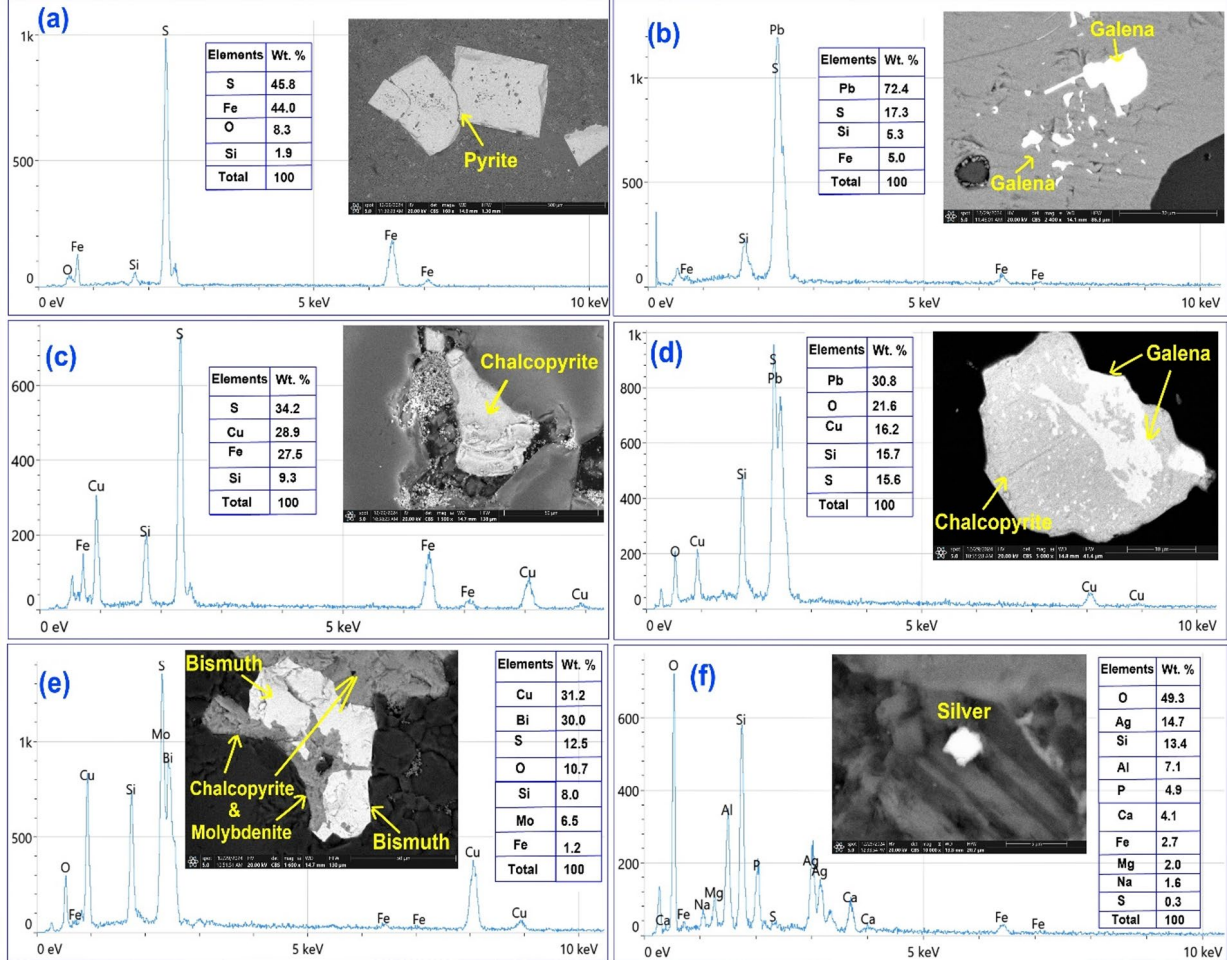
BSE image and EDX spot analysis of: (**a**) Pyrite in monzogranite. (**b**) Galena in metavolcanics. (**c**) Chalcopyrite in monzogranite. (**d**) Galena and chalcopyrite in talc carbonate. (**e**) Bismuth, chalcopyrite and molybdenite in monzogranite. (**f**) Silver in talc carbonate rocks.

**Fig. 13:**
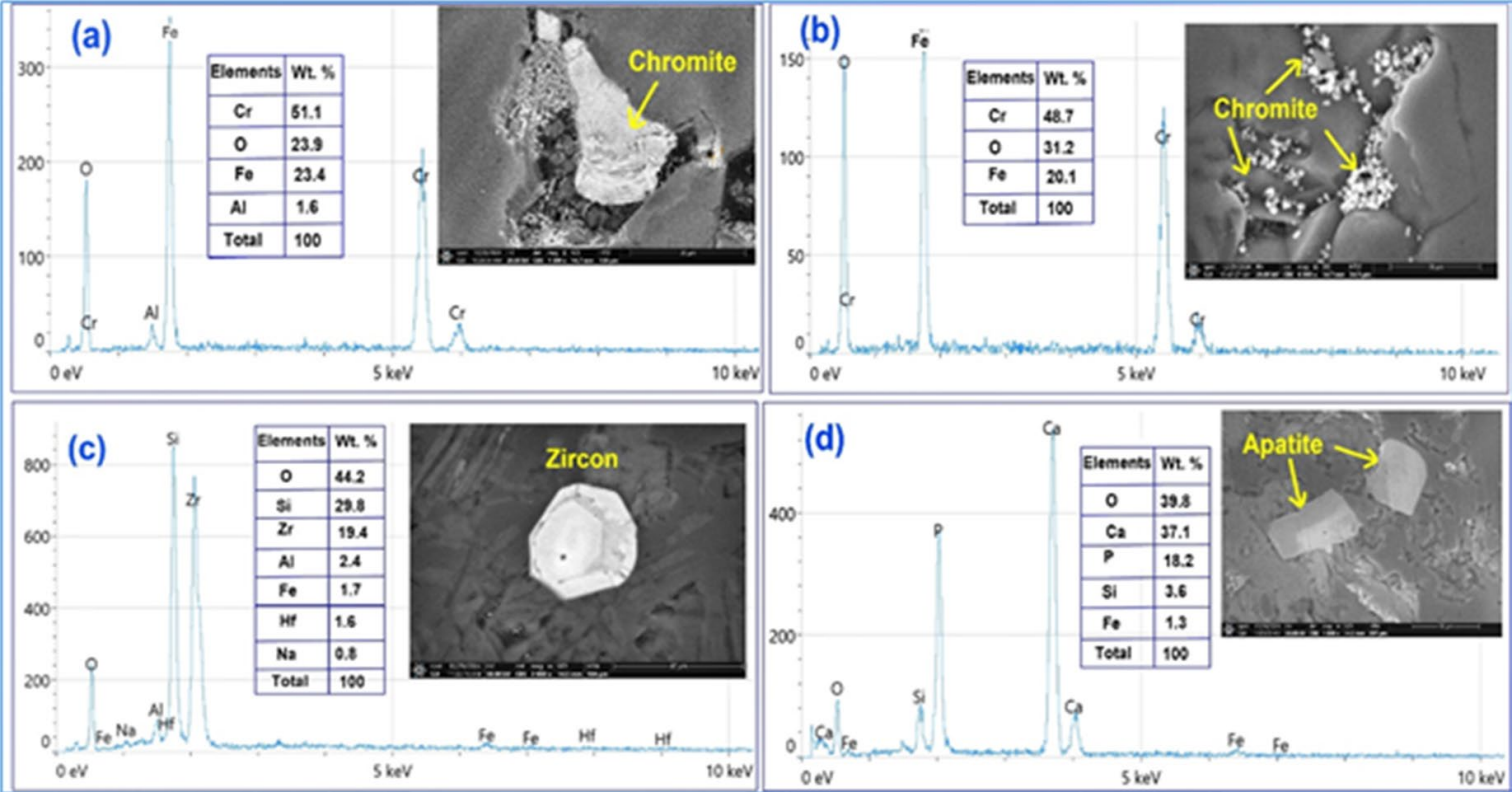
BSE image and EDX spot analysis of: (**a**, **b**) Chromite in talc carbonate rocks. (**c**, **d**) Zircon and apatite in monzogranite.

Scanning Electron Microscopy (SEM) and Energy Dispersive X-ray (EDX) analyses were performed on polished thin sections, enabling the definitive identification of mineral phases within the ophiolitic, metavolcanic, and granitic units of the study area. Microchemical analysis by EDX confirmed a diverse mineral assemblage, including pyrite, galena, chalcopyrite, silver, bismuth, chromite, zircon, apatite, and molybdenite (Figs. 12 and 13). EDX analysis of pyrite crystals within the monzogranite (Fig. 12a) revealed prominent peaks of sulfur (45.8%) and iron (44%), along with minor amounts of oxygen (8.3%) and silicon (1.9%). In metavolcanic rocks, galena crystals (Fig. 12b) showed high peaks of lead (72.4%) and sulfur (17.3%), accompanied by small quantities of silicon (5.3%) and iron (5%). Chalcopyrite crystals in the monzogranite (Fig. 12c) exhibited strong peaks of sulfur (34.2%), copper (28.9%), and iron (27.5%), with a minor silicon content (6.3%).

In talc-carbonate rocks, EDX analysis identified both galena and chalcopyrite crystals (Fig. 12d), characterized by high peaks of lead (30.8%), oxygen (21.6%), and copper (16.2%), along with smaller amounts of silicon (15.7%) and sulfur (15.6%). Within the same monzogranite, bismuth and molybdenite crystals (Fig. 12e) displayed prominent peaks of copper (31.2%), bismuth (30%), and sulfur (12.5%), with minor oxygen (10.7%), silicon (8%), molybdenum (6.5%), and iron (1.2%). Silver crystals in talc-carbonate rocks (Fig. 12f) showed major peaks for oxygen and silver, with traces of Si, Al, P, Ca, Fe, Mg, Na, and S. Chromite crystals occurring in talc-carbonate rocks (Fig. 13a,b) were marked by high peaks of chromium (48.7–51.1%), oxygen (23.9–31.2%), and iron (20.1–23.4%), along with a minor aluminum content (1.6%). Zircon crystals in the monzogranite (Fig. 13c) exhibited strong peaks of oxygen, silicon, and zirconium, with small amounts of Al, Fe, Hf, and Na. Finally, apatite crystals from the monzogranite (Fig. 13c) displayed prominent peaks of oxygen, calcium, and phosphorus, accompanied by trace amounts of silicon and iron.

### Mineralization and alteration types

The mere presence of ore minerals does not, by itself, define an economic mineralization system. In the Gabal Harga Zarqa area, the identified mineral assemblage (pyrite, galena, chalcopyrite, silver, bismuth, chromite, molybdenite, zircon, apatite) must be evaluated in the context of host lithology, hydrothermal alteration, and structural controls to determine the possible deposit types.

#### Host lithologies and their metallogenic affinities

Three main rock units host the mineralization are recorded in the studied area; (a) Serpentinite and talc-carbonate rocks (ophiolitic ultramafics) – these host chromite and, to a lesser extent, bismuth and base-metal sulfides along shear zones, (b) Metabasalts (metavolcanic rocks of the Gerf ophiolite) – these contain disseminated pyrite, chalcopyrite, and galena, locally associated with quartz-carbonate veins, (c) Syn-tectonic monzogranite – this intrusive body carries molybdenite, zircon, apatite, and vein-type chalcopyrite-galena-silver mineralization, especially near its contacts with metavolcanics.

#### Alteration types and their spatial distribution

Field and petrographic observations reveal three distinct alteration assemblages:

Talc-carbonate alteration (after serpentinite): widespread in the eastern part of the study area, characterized by replacement of serpentine and olivine by talc + magnesite/dolomite ± quartz. This alteration is typical of ophiolitic settings and is spatially associated with chromite pods and minor sulfide mineralization. Greenschist-facies metamorphic alteration (in metabasalts): chlorite + epidote + calcite + sericite, overprinting the original igneous assemblage. This regional alteration is not directly ore-related but enhances permeability for later hydrothermal fluids. Hydrothermal vein alteration (cross-cutting all units): quartz + carbonate ± sericite ± chlorite veins, often with sulfides (pyrite, chalcopyrite, galena) and locally native silver and bismuth. This alteration is structurally controlled and is the most promising for precious and base metal mineralization.

#### Deposit types and genetic interpretation

Based on the host rocks, alteration styles, and mineral assemblage, at least three deposit types can be proposed for the Gabal Harga Zarqa area in Table 1.

**Table 1:** The host Lithology and their alterations associated to structure control.

Deposit type	Host lithology	Key minerals	Alteration	Structural control
Podiform chromite	Serpentinized peridotite (talc-carbonate)	Chromite	Talc-carbonate, serpentinization	Primary magmatic segregation, later sheared
Polymetallic vein (Cu-Pb-Zn-Ag ± Au)	Metabasalts, monzogranite, and contact zones	Chalcopyrite, galena, pyrite, silver, bismuth	Quartz-carbonate-sericite veins	NW-SE sinistral shear zones (D3)
Molybdenite-bearing granite-related	Monzogranite (syn-tectonic)	Molybdenite, chalcopyrite, pyrite, zircon, apatite	Weak sericitization, quartz veining	Magmatic-hydrothermal, fracture-controlled

The polymetallic vein type is the most significant for future exploration. The presence of silver, bismuth, and copper sulfides, together with artisanal mining activity (bright pixels in remote sensing images, Figs. 6a and 8a), strongly suggests that hydrothermal fluids migrated along the NW-SE trending sinistral faults (D3 phase) and precipitated sulfides in extensional quartz veins. This setting is analogous to orogenic gold-base metal deposits known elsewhere in the Allaqi-Heiani suture^4,76^. Although gold was not directly analyzed by SEM-EDX in this study, its presence is inferred from the artisanal workings and from regional analogues. The podiform chromite in talc-carbonate rocks represents a common ophiolitic mineralization, but its economic potential is limited due to the small, deformed pod geometry. The molybdenite in the monzogranite suggests a possible granite-related Mo-Cu affinity, though grades are likely low without further enrichment.

#### Linking mineralization to the regional tectonic framework

The Allaqi-Heiani suture is a well-known gold- and base-metal-bearing belt^8,76^. In the Harga Zarqa area, mineralization is not randomly distributed; it is concentrated along two structural levels: (1) the contact between the Gerf ophiolitic nappe and the underlying metavolcanics, and (2) the NW-SE sinistral fault systems that cut both the monzogranite and the metavolcanics. These faults acted as conduits for hydrothermal fluids during the late-orogenic (D3) transpressive regime (640–560 Ma). Therefore, the area hosts a polymetallic (Cu-Pb-Zn-Ag ± Mo ± Au) vein system superimposed on an ophiolitic chromite occurrence. This dual metallogenic signature is typical of the final stages of accretionary orogens in the Arabian-Nubian Shield.

## Defomartion history of Gabal Harga Zarga klippen

Structural analyses suggest that the region has undergone a polyphase deformation history, encompassing at least two significant events. The first event, D1, involved regional shortening from N–S to NNE–SSW, resulting in the formation of SSW-verging folds and NNE-dipping thrusts. The second event, D2, was characterized by ENE–WSW shortening, which produced NNW–SSE oriented folds in the central and eastern sections of the Allaqi-Heiani belt and reactivated older thrusts, leading to oblique-slip reverse fault movements.

**Fig. 14:**
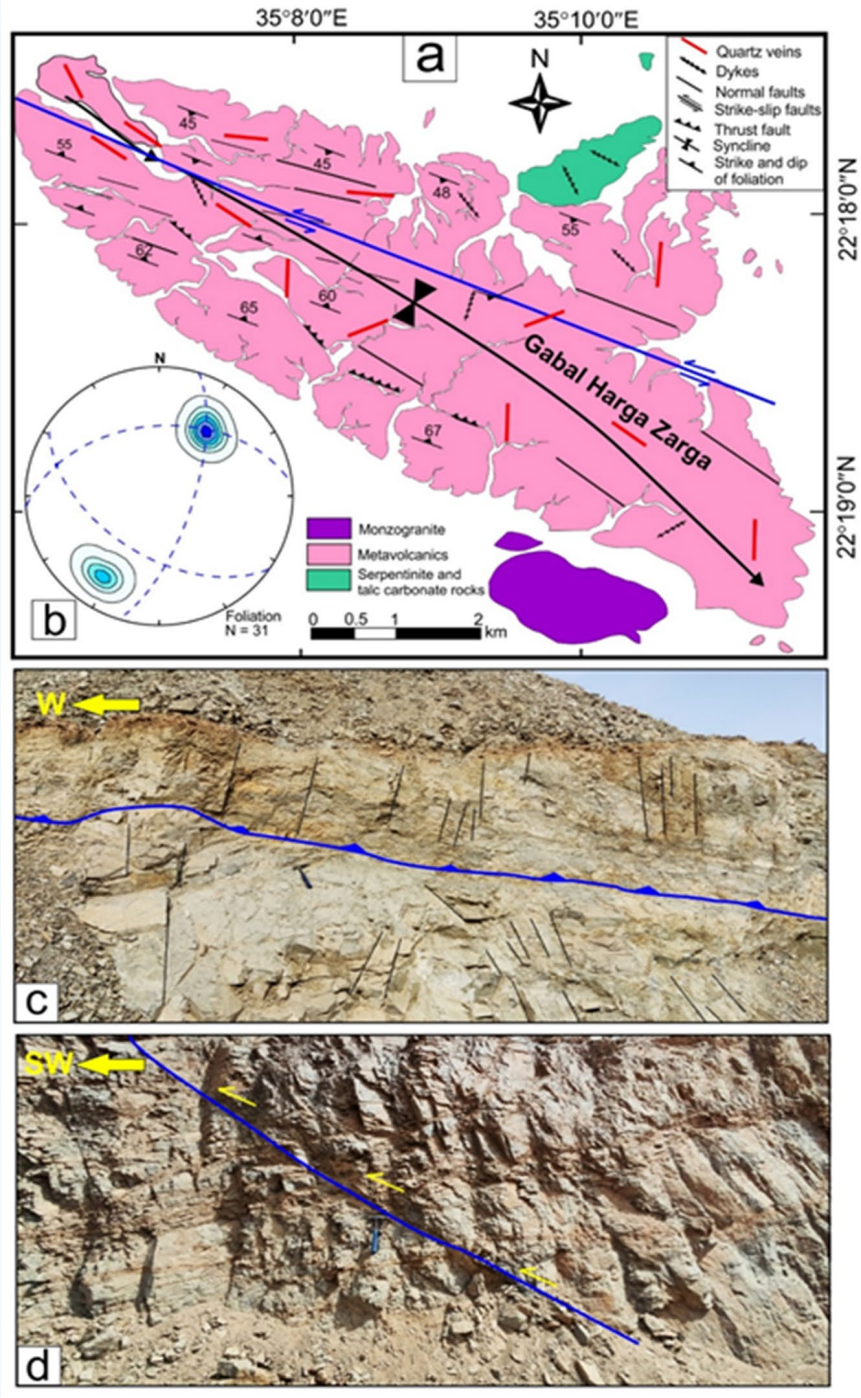
Field structure shows: (**a**) Structural map of Gabal Harga Zarga. (**b**) Stereonet showing the attitude of foliation in Gabal Harga Zarga, contours: 0, 5, 10, 15, 20. (**c**) and (**d**) thrust faults with top-to-SSE sense movement in Gabal Harga Zarga metavolcanics.

Gabal Harga Zarga, also known as Hadal Darjah or Hagar Zarqa, represents an ophiolitic mélange klippe situated in the central-western region of the mapped area, positioned to the north of Gabal Heiani and to the south of Gabal Mansur Diab. This geological formation is characterized by its folding into an asymmetrical syncline that trends in a northwest-southeast direction, with its axial plane exhibiting a dip of approximately 75° towards the southwest (Fig. 14a,b). The composition of the Gabal Harga Zarga klippe is predominantly made up of metabasalts and meta-andesites, accompanied by metasedimentary rocks and a smaller presence of serpentinites^55^.

The deformation history of the Gabal Harga Zarga region commenced during the D1 deformation phase, characterized by the collision of the Eastern Desert and Gabgaba terranes. This event occurred in the context of an early phase of north-south shortening, which significantly influenced the geological evolution of the area^8,12,55,61,77–79^. The early shortening event, designated as D1, is associated with the oblique accretion of the island arc during the period of 740 to 680 Ma. This event is characterized by the complex stacking and imbrication of the Pan-African cover, which exhibits a directional trend from south-southwest to north-northwest. Evidence for the NNW–SSE shortening in the Allaqi-Heiani belt is provided by the presence of large-scale folds and thrust faults that exhibit a top-to-SSE movement (Fig. 14c,d). The ophiolitic units record D1 fabrics (S1 and F1), such as E-W fold interference patterns and E-W thrust. The NW-trending asymmetrical syncline in Gabal Harga Zarga klippen is formed during D1. The Gabal Harga Zarga klippen underwent folding into coaxial synclinal structures that generally trend NW-SE (Fig. 14a,b). The axial plane of the Harga Zarga syncline exhibits a SW dip, indicating an easterly vergence, which implies the presence of orogenic stress originating from the east.

The development of the Hamisana shear zone during the second deformation phase (D2) was primarily influenced by shortening along the E-W axis. This tectonic activity resulted in the establishment of schistosity (S2) and the formation of upright and isoclinal folds (F2) that exhibit a north-to-northwest alignment. During D2, the geological structures S1 and F1 experienced overprinting due to E-verging thrusts, resulting in their subsequent overturning and the development of recumbent and tight folds, designated as F2. This phase of folding was concluded during D3, which is dated between 640 and 560 million years ago, marking a significant transition in the tectonic landscape.

The culmination of the folding processes during D3 was characterized by the establishment of dextral strike-slip shear zones oriented towards the north. This tectonic activity facilitated the formation of folds trending east-west, alongside the emergence of very tight folds that trend northward (F3). The transpressive stresses linked to the D3 phase within the Hamisana shear zone, resulting from the dextral couple, have led to the formation of folds that dip at moderate angles toward the northwest and north-northwest. The Hamisana shear zone exhibits dextral shearing, which has resulted in the division of the Sol Hamed-Onib-Heiani-Allaqi suture into two distinct branching planes, characterized by displacements reaching as much as 50 km. The two resulting branches from this shear zone consist of sinistral strike-slip shear zones that extend toward the northwest, identified as the Allaqi-Heiani suture, and dextral strike-slip shear zones that trend to the northeast, referred to as the Sol Hamed-Onib suture. The differentiation between these two types of shear zones underscores the complexity of tectonic interactions occurring within the suture system.

The geological landscape of the Gabal Harga Zarga klippen, located in the western domain, has been notably influenced by sinistral strike-slip faults that trend northwest to north-northwest (Fig. 14a). The activity of these faults during the D3 phase underscores the ongoing tectonic evolution and the structural complexities inherent in Gabal Harga Zarga (Table 2).

**Table 2:** Summary of deformation history of Gabal Harga Zarga area.

Aspect	Details
Polyphase Deformation	Region experienced at least three main deformation events: D1, D2, and D3.
D1 event	*Timing*: 740–680 Ma *Type*: Regional shortening *Direction*: N–S to NNE–SSW *Structures Formed*: SSW-verging folds, NNE-dipping thrusts, large-scale folds/thrust faults with top-to-SSE movement *Associated Fabrics*: S1 and F1 (E–W interference patterns and E–W thrusts) *Geological Setting*: Collision of Eastern Desert and Gabgaba terranes; oblique accretion of island arc *Effect on Gabal Harga Zarga*: Formation of NW-trending asymmetrical syncline; folding with SW-dipping axial plane (easterly vergence)
D2 event	*Timing*: After D1, before D3 *Type*: ENE–WSW shortening *Direction*: ENE–WSW *Structures Formed*: NNW–SSE folds (central/eastern Allaqi-Heiani belt), upright and isoclinal folds (F2), schistosity (S2), overprinting of S1/F1, recumbent/tight folds (F2), reactivated older thrusts, oblique-slip reverse faults *Key Zone*: Development of Hamisana shear zone *Effect*: Overturned structures, complex fold geometries, established new fold generations and schistosity patterns
D3 event	*Timing*: 640–560 Ma *Type*: Dextral strike-slip shearing (transpression) *Direction*: Northward *Structures Formed*: E–W trending folds (F3), very tight northward folds, moderate NW/NNW dipping folds *Shear Zones*: Hamisana shear zone splits suture into two: sinistral (NW, Allaqi-Heiani suture), dextral (NE, Sol Hamed-Onib suture), with displacements up to 50 km *Fault Activity*: Sinistral strike-slip faults (NW–NNW in Gabal Harga Zarga), dextral strike-slip shear zones (NE)
Focus area	*Main Locality*: Gabal Harga Zarga (also Hadal Darjah or Hagar Zarqa) *Type*: Ophiolitic mélange klippe *Location*: Central-western mapped region, north of Gabal Heiani, south of Gabal Mansur Diab *Fold Geometry*: Asymmetrical syncline, NW–SE trend, axial plane dipping ~ 75° SW *Vergence*: Easterly vergence, orogenic stress from east
Lithology	*Main Rocks*: Metabasalts, meta-andesites *Other Components*: Metasedimentary rocks, minor serpentinites
Structural evolution	*Initial Setting*: Early north-south shortening, complex stacking/imbrication of Pan-African cover (SSW–NNW trend) *Folding*: Coaxial synclinal structures (NW–SE trend), SW-dipping axial planes *Fabrics*: D1 fabrics identified (S1, F1) *Overprinting*: D2 overprints D1, leads to upright, isoclinal, recumbent, tight folds, schistosity *Shear Zone Dynamics*: D3 establishes dextral shear, tightens folds, divides sutures, new faults/folds form
Tectonic zones & sutures	*Hamisana Shear Zone*: Dextral shearing, splits suture into two: sinistral (Allaqi-Heiani), dextral (Sol Hamed-Onib) *Branching Planes*: Sinistral strike-slip (NW, Allaqi-Heiani suture), Dextral strike-slip (NE, Sol Hamed-Onib suture) *Displacement*: Up to 50 km *Tectonic Complexity*: Multiple interacting faults, folding, and suture segmentation
Geological impact	*Ongoing Evolution*: Active tectonic processes, structural complexity *Fault Influence*: Sinistral strike-slip faults (NW–NNW trend) in western Gabal Harga Zarga *Landscape Result*: Complex fold/fault patterns, partitioned sutures, significant tectonic and structural diversity
References	^8,12,55,61,77–79^

## Discussion

This study presents a significant advancement in the geological understanding of the Gabal Harga Zarga (Harga Zarqa) region by integrating remote sensing, petrographic, and structural analysis to unravel the area’s complex lithological and tectonic framework. The findings have several important implications for regional geology, mineral exploration, and methodological approaches in remote terrains.

One of the major contributions of this work is the revelation of the area’s lithological heterogeneity. Contrary to earlier regional maps that classified the region as a single metavolcanic block, the integrated approach employed here identifies a mosaic of lithologies, including serpentinite, talc-carbonate, metabasalt, and monzogranite. This improved lithological discrimination is crucial, as it directly impacts the interpretation of tectonic processes and the assessment of mineralization potential. The identification and spatial distribution of these rock units, supported by both remote sensing and field validation, highlight the effectiveness of multi-sensor datasets (PRISMA, Sentinel-2, and PlanetScope) in capturing compositional and structural variability in complex terrains.

The study also sheds light on the intricate structural evolution of the area, identifying multiple deformation phases (D1–D3) that have shaped the present-day configuration. The recognition of NW-SE trending sinistral faults and their role in controlling hydrothermal fluid flow and mineralization is particularly noteworthy. These structural elements have not only influenced lithological boundaries but also served as conduits for mineralizing fluids, resulting in polymetallic vein systems. The documentation of this tectonic-structural control aligns the Harga Zarqa region with other mineralized belts within the Arabian-Nubian Shield, reinforcing its exploration potential for base and precious metals.

The successful application of advanced remote sensing techniques, especially the use of hyperspectral PRISMA data, demonstrates a replicable workflow for geological mapping in remote and inaccessible regions. The integration of remote sensing with field and laboratory analysis ensures robust lithological and mineralogical interpretations, reducing reliance on extensive ground surveys and offering a cost-effective model for similar geological settings elsewhere.

Furthermore, the mineralogical investigations provide new insights into the region’s metallogeny. The identification of ore minerals such as pyrite, galena, chalcopyrite, silver, bismuth, chromite, zircon, apatite, and molybdenite across different rock units suggests a dual metallogenic system, combining features of ophiolitic chromite and orogenic/hydrothermal polymetallic vein deposits. While direct evidence for economic gold mineralization remains limited, the presence of artisanal mining and regional analogs point to promising future targets.

In summary, this study not only fills a significant knowledge gap regarding the geology of Egypt’s extreme southeastern desert but also provides a methodological blueprint for integrated geological investigations in similarly challenging environments. The outcomes are directly relevant to mineral resource evaluation and sustainable development, and they offer a foundation for future research focused on both detailed mineral exploration and tectonic reconstruction of the Arabian-Nubian Shield.

## Conclusion

This study represents the geological investigation of the Harga Zarga region, an exceptionally remote area in the southernmost reaches of Egypt’s Eastern Desert. For the first time over the study area, multi-sensor remote sensing datasets—specifically Sentinel-2, PlanetScope, and PRISMA hyperspectral data—were integrated with rigorous structural analysis and petrographic studies. We have produced an updated and verified geological map. This multifaceted approach provides a definitive lithological and structural framework, essential for evaluating the region’s mineral potential, with a particular focus on gold mineralization. Our study concluded the following:

1. The Harga Zarqa mountain is lithologically complex and not as straightforward as previously depicted in lithological maps, which represented it as a single metavolcanic block. Our integrated approach has shown that the area is mainly composed of heterogeneous metavolcanics and serpentinite-talc blocks, particularly in the eastern part of the study area. These serpentinites sometimes form long rock exposures that align with the region’s overall NW-SE tectonic orientation.
2. According to field observations and petrographic analysis, the study area is classified into three main rocks: talc carbonates, metabasalts, and syn-tectonic granites. Ophiolitic rocks are represented by talc-carbonate alteration products of serpentinite rocks. It is characterized by equal ratios of talc and carbonate. Metavolcanic rocks are represented by metabasaltic rocks, composed of essentially plagioclase and pyroxene crystals. Also, these rocks contain secondary minerals, such as carbonates and chlorite. Muscovite-granite consists mainly of K-feldspars, plagioclase, quartz, biotite, and muscovite, while epidote and iron oxides are the main secondary minerals.
3. The western domain’s Gabal Harga Zarga Klippen’s geological landscape is significantly shaped by sinistral strike-slip faults that trend from northwest to north-northwest. These faults’ activity throughout the D3 period highlights Gabal Harga Zarga’s structural complexity and continuous tectonic evolution.
4. The identification of base‑metal sulfides (chalcopyrite, galena), precious metals (silver), and accessory minerals (zircon, apatite, molybdenite) in the ophiolitic, metavolcanic and granitic rocks, combined with evidence of artisanal mining activity (bright pixels in remote sensing images), highlights the area’s potential for gold, silver, copper, lead, and molybdenum mineralization. This study thus provides a baseline for future exploration programs.
5. This research not only fills a critical gap in the geological knowledge of Egypt’s extreme south‑eastern desert but also demonstrates a cost‑effective, multi‑scale methodology that integrates remote sensing with classical field and laboratory techniques. These results are directly applicable to mineral resource assessment and sustainable development in similar remote terrains worldwide.

## Data Availability

The datasets used and/or analyzed during the current study are available from the corresponding author upon reasonable request.
